# Tracking GAD-specific T cell expansions in type 1 diabetes by intradermal GAD-alum challenge

**DOI:** 10.1172/jci.insight.203119

**Published:** 2026-09-01

**Authors:** Stephanie J. Hanna, Emma J.S. Robinson, Terri C. Thayer, Maki Nakayama, Laurie Landry, Robert Andrews, Garry Dolton, Joanne Davies, Evangelia Williams, James A. Pearson, Andrew K. Sewell, Parth Narendran, David C. Wraith, Alexandra Howell, Philippa Young, Mary Hart, Anton Lindqvist, F. Susan Wong, Tim I.M. Tree, Colin M. Dayan, Danijela Tatovic

**Affiliations:** 1 Division of Infection and Immunity, Cardiff University School of Medicine, Cardiff, United Kingdom.; 2 Division of Immunology and Immunotherapy, University of Birmingham, Birmingham, United Kingdom.; 3 Center for Infectious Diseases and Immunology, Rochester General Hospital Research Institute, Rochester, New York, USA.; 4 Barbara Davis Center for Childhood Diabetes, University of Colorado, Denver, USA.; 5 Department of Immunobiology, School of Immunology & Microbial Sciences, King’s College London, London, United Kingdom.; 6 Breast Test Wales, Public Health Wales, Cardiff, United Kingdom.; 7 Department of Radiology, University Hospital of Wales, Cardiff, United Kingdom.; 8 Diamyd Medical AB, Stockholm, Sweden.

**Keywords:** Autoimmunity, Immunology, Diabetes, T cell receptor, T cells

## Abstract

Identifying and monitoring autoreactive T cells that drive β cell destruction remains a major obstacle to developing effective immunotherapies for type 1 diabetes (T1D). These cells are extremely rare in peripheral blood and cannot be accessed directly from the pancreas. We used intradermal injection of glutamic acid decarboxylase (GAD)-alum to recruit GAD-specific T cells to accessible sites in the skin and skin-draining lymph nodes (LNs), sampled by skin suction blisters and ultrasound-guided LN aspiration. Peripheral blood samples obtained before GAD injection were restimulated with GAD in vitro to detect reactive CD4^+^ T cells. Single-cell RNA sequencing (scRNA-seq) followed by re-expression of selected T cell receptors (TCRs) confirmed antigen specificity. Up to 70% of T cells at the skin injection site were clonally expanded and 4 of 14 (28%) re-expressed TCRs were GAD reactive. In LNs, 1 of 14 (4%) clonally expanded TCRs was GAD reactive, representing approximately 0.08% of all T cells. GAD-reactive cells across compartments displayed Th1- and Th17-associated transcription signatures. These results demonstrate that intradermal autoantigen challenge and scRNA-seq enable direct identification and molecular profiling of autoreactive T cells in vivo. This minimally invasive approach provides a powerful platform for tracking antigen-specific T cells to monitor disease activity and evaluate immune interventions in T1D.

## Abstract 2

In Type 1 diabetes we found T cells normally only present in the pancreas by injecting pancreas protein under the skin.

## Introduction

The licensing of teplizumab to delay the onset of type 1 diabetes (T1D) has demonstrated the potential of immune therapies to postpone insulin dependence (1). To accelerate the development of disease-modifying therapies, it is essential to develop techniques that can monitor how interventions affect the pathogenic T cell response that drives β cell destruction. However, tracking antigen-specific T cells in T1D is extremely challenging due to their low frequency in peripheral blood (~1:10,000) (2–4) and the inaccessibility of the pancreas and its draining lymph nodes (LNs) in living donors. Several approaches have been used to overcome this limitation, including isolation of tetramer-positive cells from the peripheral blood (5, 6), in vitro activation assays to enrich antigen-specific cells (7), and T cell receptor (TCR) sequencing of islet-infiltrating lymphocytes from deceased donors followed by re-expression in cell-based systems to confirm specificity (8). Each has significant drawbacks. Tetramer-based detection is limited by low frequencies, some non-specific binding, and the need to predefine epitopes and HLA restriction. ELISPOTs restrict phenotypic analysis to cytokine-secreting cells, and prolonged in vitro culture can bias clone selection and distort the in vivo phenotype. Flow and mass cytometry have provided important insights into cell phenotypes in T1D (9, 10), but the limited panel sizes restrict discovery of novel biomarkers. There is therefore a clear need for methods that combine confident identification of disease-relevant antigen-specific T cells with complete, unbiased phenotypic profiling that faithfully reflects their in vivo status. Single-cell RNA sequencing (scRNA-seq) is ideally suited to this, with early studies identifying predictive biomarkers in individuals who progress to T1D (11) and defining transcriptional pathways specific to islet-antigen-reactive lymphocytes (7).

Developing approaches that allow reliable profiling of antigen-specific T cells in T1D will be key to monitoring and optimizing how immunotherapies modify disease activity, identifying at-risk individuals and matching them to the most appropriate therapeutic strategy (12–15).

We recently reported that intradermal injection of a proinsulin peptide (C19-A3) conjugated to gold nanoparticles (GNPs) induced marked clonal expansion of T cells at the injection site, with over 50% of expanded clonotypes specific for either proinsulin or the GNP core particle (16). The frequency was approximately 1000-fold higher than that seen in the peripheral blood. We also showed that intradermal injection of recall antigens such as tuberculin purified protein derivative (PPD) led to accumulation of antigen-specific cells in skin-draining LNs within 3–5 days (17, 18). These findings led us to hypothesize that diabetes autoantigen-specific T cells could be recruited to accessible sampling sites by intradermal injection of whole autoantigens, enabling their isolation and characterization in vivo (18).

Glutamic acid decarboxylase 65 (GAD) is a major autoantigen targeted by both T cells and B cells in T1D (19). Here, we combine scRNA-seq with intradermal injection of GAD-alum to identify and profile GAD-specific T cells in people with T1D using 3 complementary enrichment strategies: (a) recruitment to the injection site, (b) enrichment in the skin-draining LNs, and (c) ex vivo activation and isolation of GAD-reactive peripheral blood CD4^+^ T cells.

## Results

### Response to intradermal GAD-alum injection.

In order to recruit GAD-specific T cells to the skin and skin-draining LNs, we enrolled participants with T1D and positive for GAD autoantibodies and gave them an intradermal injection of GAD-alum in the deltoid region (Supplemental Tables 1–3; supplemental material available online with this article; https://doi.org/10.1172/jci.insight.203119DS1). All participants who received intradermal GAD-alum developed an immediate local skin reaction characterized by a circular erythematous area approximately 2–3 cm in diameter. The erythema resolved within 45–60 minutes under observation. A delayed asymptomatic erythematous skin induration of similar size then appeared at the injection site 2 days later and resolved completely within 15 days (Supplemental Figure 1).

### Response to intradermal GAD-alum in the skin and skin-draining LNs.

Based on our previous work, we hypothesized that intradermal injection of GAD-alum would recruit clonally expanded T cells to the skin and skin-draining LNs (18). The rapid onset of local skin responses suggested recruitment of pre-existing antigen-experienced T cells, rather than de novo clonal expansion following antigen exposure and we hypothesized that they might also traffic to the draining LNs. By examining cells in the skin and draining LNs after intradermal GAD challenge, we aimed to enrich for GAD-specific cells by recirculation into LNs and be able to capture their in vivo phenotype without requiring in vivo (or ex vivo) expansion. Such clonal expansions would have occurred prior to intervention, in response to endogenous GAD.

Skin suction blisters were obtained from the injection sites of 3 participants with T1D (Donors C, D, and E), 5–13 days after GAD-alum administration. The fluid from the suction blisters was aspirated and the cells in the fluid were subjected to scRNA-seq. scRNA-seq–derived TCR sequences were obtained from all 3 donors and gene expression (GEX) data were generated from blisters from Donors D and E (Figure 1A and Supplemental Tables 4–6). LN aspirates were collected before and after GAD-alum injection from 5 donors. We refer to these samples as LN_Pre and LN_Post. A total of 67,780 LN cells and 2620 blister cells passed quality control (QC) after sequencing and were used in this analysis (Supplemental Tables 7–9). We annotated cell clusters based on expression of curated lists of genes defining canonical T cell subsets (Supplemental Figure 2). All blisters with GEX information demonstrated T cell infiltrations (Figure 1A and Supplemental Figures 3 and 4). LNs contained abundant CD4^+^ T cells, smaller CD8^+^ T cell proportions, and substantial B cell fractions (Figure 1A and Supplemental Figure 4). Clonally expanded T cells, defined by identical paired TCRα and TCRβ sequences, were mapped onto Uniform Manifold Approximation and Projection (UMAP) plots (Figure 1B) (TCRs were compared at the amino acid level. To be defined as a clone, CDR3 sequences were identical, as were V and J gene usage. Amino acid changes outside of the CDR3 were permissible. Morisita-Horn indexes were calculated from the Seurat objects after QC).

Consistent with our previous findings (18, 20), we expected GAD-specific T cells to be enriched among clonally expanded populations in the LNs. Clonal expansions were present both before and after intradermal GAD-alum injection, representing approximately 0.1%–2.5% of all T cells, with no significant change in abundance (Figure 2, A and B, and Supplemental Figure 5).

Donors C, D, and E all demonstrated substantial clonal expansions in skin blisters (~20%, 5%, and 45% of all T cells, respectively, exceeding those observed in LNs) (Figure 2B). Donor E also showed some evidence of antigen-presenting cells clustering with T cells, possibly reflecting ongoing physical interactions and antigen recognition in situ (21).

Uninjected control blisters were collected from 3 donors with T1D who did not receive GAD-alum (Supplemental Tables 10–12). The total number of TCR-bearing cells was significantly higher in GAD-injected compared with control blisters (*P* < 0.05, 1-tailed *t* test), although the increase in clonally expanded TCRs did not reach statistical significance (Figure 2, C and D). The total number of cells recovered from the GAD-injected blisters was also increased compared with controls (data not shown).

### Re-expression of TCRs to confirm GAD specificity.

The large clonal expansions observed in the skin strongly suggested an antigen-specific response (18), which we sought to confirm by re-expressing the TCRs in a cell-based system. We selected TCRs with paired α and β chains representing the highest clonal expansion, including both CD4^+^ and CD8^+^ clonotypes where possible (Tables 1 and 2) and expressed them in the 5KC reporter line (22). TCR transductants were first screened for activation by GAD-alum presented by autologous lymphoblastoid cell lines (LCLs) and those showing a significant increase in NFAT reporter signal (data not shown) were subsequently tested against GAD-expressing LCLs. This analysis demonstrated that 4 of 14 (29%) of the blister-derived TCRs were GAD specific, including at least one GAD-reactive TCR from each donor as indicated by increased fluorescence in the NFAT reporter assay (Figure 3A, Tables 1 and 2, and Supplemental Tables 13–22).

### Re-expression of LN TCRs identified GAD-specific T cells.

From the LN clonal expansions, we re-expressed the most abundant postinjection TCRs and assessed their GAD reactivity in vitro using the 5KC reporter system. TCR transductants were initially screened with GAD-alum, and those showing significant NFAT reporter response (not shown) were subsequently screened using GAD-expressing LCLs. Of the 24 TCRs re-expressed from 22 T cell clones studied (2 T cell clones each expressing 2 TCRα chains), one clonotype (~4% of those tested) demonstrated GAD specificity in postinjection LN samples (Figure 3A and Tables 1 and 2) and was designated TCR B_post_GAD_LN-5.

Shared clonally expanded TCRs detected in both LN_Post and LN_Pre samples from the same donor were not GAD specific (Tables 1 and 2). All confirmed GAD-specific TCRs derived from CD4^+^ T cells, while none of the 11 CD8^+^ T cell TCRs tested exhibited GAD reactivity (Tables 1 and 2). Alum alone did not induce NFAT reporter activation in 5KCs expressing these TCRs (data not shown). When mapped back onto the UMAP plot, cells expressing the same GAD-specific TCR clonotype exhibited highly similar gene expression profiles, while expression patterns differed between distinct GAD-specific clonotypes (Figure 3B).

### T cell phenotypes in the LN and blister.

LN T cells had phenotypes consistent with recirculating T cell populations and across all samples, clonally expanded cells were enriched within 2 major clusters: CD8^+^ T cells expressing *IFNG*, and CD4^+^ T cells expressing high levels of the Th1- and Th17-associated chemokine receptors *CCR6* and *CXCR3* (Figure 1 and Supplemental Figure 2). We focused particularly on Th17.1 cells, given our previous observations that reductions in this subset predict response to ustekinumab in T1D (20). However, in the absence of ex vivo stimulation, these CD4^+^ T cells did not express *IL17A* (Figure 4).

### Phenotypes of GAD-specific cells in the skin.

Because a high proportion of the re-expressed clonally expanded TCRs from the blisters were GAD specific, we compared all blister T cells (with a defined TCR and not associated with antigen-presenting cells) from the GAD-injected participants to those from control donors. Following GAD injection, T cells showed upregulation of mitochondrial and ribosomal genes, consistent with increased cellular activation. TRRUST analysis indicated that these genes were regulated by transcription factors from the RFX family (Figure 5A and Supplemental Table 23). We next examined canonical T cell subset markers and found substantial interdonor variability. Donor D exhibited expression of *IL22*, *CXCR3*, *CCR6*, and *LTB*, genes associated with Th17 cells and autoimmune responses (23), whereas this pattern was not observed in Donor E (Supplemental Figure 6).

The above analysis was limited by the small number of cells in the control blisters. To further characterize phenotypes associated with GAD-specific T cells, we compared all post-GAD Alum blister T cells within conventional CD4^+^ T cell clusters (i.e., excluding those associated with monocytes) to CD4^+^ T cells from pre-GAD LNs. The top DEGs included *IL22*, *CXCR6*, and *CD26* as well as activation markers such as *TNFRSF18* (GITR) (Supplemental Table 24). These changes were primarily driven by high expression in Donor D, who contributed more conventional CD4^+^ T cells than Donor E (Supplemental Figure 7). TRRUST analysis identified NF-κB, RelA, and SP1 (which regulates *TBET* expression; ref. 24) as the top 3 transcription factors driving the observed gene expression profile in blister T cells (Figure 5B)

### Phenotypes of T cells in the LNs following intradermal GAD-alum.

In addition to sampling suction blister fluid, we also performed ultrasound-guided fine-needle aspiration (FNA) of the skin-draining LNs before and after intradermal injection of GAD-alum. We examined changes in clonally expanded T cells following GAD-alum injection, hypothesizing that recruitment of GAD-specific T cells into the clonally expanded T cell clusters would generate a detectable transcriptional signature. Focussing first on CD4^+^ clonally expanded T cells, differential expression analysis of post-GAD samples revealed upregulation of ribosomal genes consistent with increased transcriptional activity, and of TXNIP. TRRUST analysis performed using Metascape (25) on upregulated genes (uncorrected *P* < 0.05) identified NF-κB, RelA, and STAT3 as the top 3 transcription factors driving the post-GAD signature (Figure 5C and Supplemental Table 25). Genes regulated by these factors included *TXNIP* and members of the TNF superfamily. When examined by donor, *TXNIP* upregulation was particularly consistent across individuals (Supplemental Figure 8). Similar results were observed when a pseudobulk analysis was performed (Supplemental Figures 9–11, and Supplemental Table 26).

We next analyzed clonally expanded cells in the *IFNG*^+^ CD8^+^ T cell cluster. Again, the most significantly upregulated genes were associated with increased activation and protein translation. TRRUST analysis identified RBMX, NF1, and FOXO1 as key transcriptional regulators of this signature (Figure 5D and Supplemental Table 27). Similar results were observed when a pseudobulk analysis was performed (Supplemental Figures 9–11, and Supplemental Table 28), although the small number of donors limited the statistical power of this approach.

### Comparison of gene expression in GAD-reactive peripheral blood T cells reveals distinctive autoantigenic phenotypes.

All participants were positive for anti-GAD autoantibodies and had T1D. We therefore hypothesized that GAD-specific T cells could be detected in peripheral blood prior to GAD challenge. To test this, we used an in vitro activation-induced maker (AIM) assay to enrich and identify GAD-reactive CD4^+^ T cells in PBMC samples collected before antigen challenge.

To this end, we performed an in vitro AIM assay to detect GAD-reactive CD4^+^ T cells prior to GAD injection. Unstimulated cells served as a negative control for unactivated cells, staphylococcal enterotoxin B–stimulated (SEB-stimulated) cells as a positive control, and buffer-stimulated control (PBMC_Ctrl) as a control for non-specific activation, or preactivated responses. A total of 693 cells passed QC from the Ctrl and GAD-stimulated wells and were included in the analysis (Supplemental Tables 29 and 30). Sorted cells formed distinct clusters, including resting cells, Tregs, 3 populations of activated central memory (TCM) cells, and 2 clusters coexpressing *IL17A* and *IFNG*, designated Th17.1s (Figure 6, A and B). As expected, cluster frequencies differed substantially between SEB-stimulated and unstimulated conditions (data not shown). The mean stimulation index of GAD versus control stimulation, corrected for initial PBMC input, was 2.2 (range 1.2–4.9). Th17.1 clusters were enriched in *IFNG*, *IL17A*, *IL17F*, and *CSF2*. Subsets designated Th17.1 *IL2*^+^ and Th17.1 *IL22*^+^ coexpressed *IL2* or *IL22*, respectively (Figure 6B). These populations were of particular interest given our previous finding that reduction in Th17.1 cells predicts response to ustekinumab in T1D (20). Compared with PBMC_Ctrl cells, PBMC_GAD cells exhibited increased expression of *IFNG*, *IL17A*, *IL17F*, *CSF2*, *IL2*, and *IL22* as well as the Th1- and Th17-associated transcription factors *STAT1* and *STAT3* (Figure 6B). Pairwise comparisons between control and GAD stimulation for each donor showed this pattern of particularly elevated *IL17A*, *IL17F*, and *IL22* expression, was consistent across all donors except Donor B, who displayed the lowest stimulation index and an insufficient number of surviving cells after scRNA-seq QC (Supplemental Figure 12). Despite these transcriptional changes, the frequency of cells within individual clusters did not increase. Differential gene expression analysis across all cells revealed PBMC_GAD cells were defined by upregulation of *IL2RA,* *IL2RB*, *LTB*, *DPP4* (CD26), and *IL22* (Figure 6B and Supplemental Table 31). TRRUST analysis identified *STAT1* and *STAT3* in the top transcription factors driving the gene expression program in GAD-reactive cells (Figure 7A). Pseudobulk analysis demonstrated similar findings, with *IL17F* and *IL22* in particular consistently upregulated in the GAD-stimulated samples (Supplemental Figures 9–11, and Supplemental Table 32).

One large clonal expansion of GAD-reactive cells was detected in Donor D (15 cells, Figure 7B). These cells predominantly exhibited a Th17.1 *IL2*^+^ phenotype and re-expression of their TCR in the 5KC reporter line confirmed GAD specificity (Tables 1 and 2, and Figure 7C). Interestingly, despite being from a different donor (Donor B), the TCRα chain of the LN-derived clone B_post_GAD_LN-5 (CAENGAGGTSYGKLTF) differed from that of the PBMC clone PBMC-1 (Donor D, CAENNAGGTSYGKLTF) by only a single amino acid. These donors shared several HLA alleles, including HLA-DRB1*04:01:01.

In addition, we also examined the phenotype of GAD-reactive cells in the peripheral blood after the injection of GAD-alum using the AIM assay, comparing their frequency and phenotype to those sampled prior to in vivo GAD-alum (Supplemental Figure 13). The percentage of GAD-reactive T cells in the peripheral blood was not altered (data not shown).

PBMC-1 GAD-specific clones were also present in the post-GAD sample, stimulated with in vitro GAD (i.e., only in the in vitro GAD-stimulated samples before and after in vivo GAD and not at all in the Ctrl stimulated wells at either time point, Supplemental Figure 13B), and predominantly retained a Th17.1 *IL2*^+^ phenotype.

Comparing abundance of clusters (again excluding Donor B who had insufficient cells), there was a significant (*P* < 0.05, paired *t* test) increase in GAD-reactive cells with a Th17.1 *IL2*^+^ phenotype and a decrease in GAD-reactive cells with a Th17.1 *IL22*^+^ phenotype (this significance did not remain after correction for multiple testing). In contrast, no changes in the Th17.1 populations were noted in the Ctrl stimulations, although the proportion of Ctrl Tregs did increase after in vivo GAD-alum.

Sharing of paired TCRs was predominantly observed between samples from the same donor (Supplemental Figure 14 and Supplemental Table 33). Overlap was detected between peripheral blood, LN, and blister samples within donors, including persistence of clonotypes across LN time points and sharing between LN and blister compartments. In contrast, sharing between donors was limited. A notably high Morisita-Horn index was observed for Donor D due to the high clonal expansion of PBMC_1 in peripheral blood samples. Higher sharing was also noted in the LNs where clones persisted between the 2 sampling time points in all 5 donors. Both blister samples with GEX information showed evidence of repertoire sharing with the same donor’s LN samples. Sharing between Ctrl PBMC samples was skewed by very low total cell numbers (cells that were activated in response to buffer components or cells that were already activated in vivo, i.e., a persistent immune response to pathogens between the sampling time points and compartments). Sharing of clones (paired TCRs) between donors was more limited (Supplemental Table 33).

Together, these findings demonstrate that GAD-specific CD4^+^ T cells can be isolated and characterized from peripheral blood, skin, and LN samples following intradermal GAD-alum administration. Across compartments, these cells displayed transcriptional features consistent with Th1 and Th17.1 phenotypes, including activation of NF-κB–, RelA-, and STAT-driven pathways. The identification of clonally expanded, GAD-reactive TCRs in both peripheral and lymphoid compartments provides new opportunities to track autoantigen-specific T cells longitudinally and to evaluate their modulation during immunotherapeutic intervention.

## Discussion

In this study, we addressed the challenge of tracking antigen-specific cells in T1D by combining intradermal injection of a diabetes autoantigen with sampling from multiple accessible sites of antigen interaction. Following intradermal GAD-alum injection, we observed striking clonal expansion of T cells in the skin, with 20%–45% of all T cells clonally expanded and up to 15 copies of individual clones. Re-expression of these TCRs confirmed that approximately 30% were GAD specific, representing approximately 1 in 10 T cells recovered from the skin injection site. In the draining LNs, approximately 2% of T cells were clonally expanded and 4% of these were GAD reactive, equivalent to 1 in 1000 LN T cells. We did not observe any trend towards increased clonal expansion after GAD treatment. Clonally expanded LN T cells also showed transcriptional changes consistent with antigen-driven activation. In peripheral blood, clonal expansions were rare, but one clonotype was confirmed GAD reactive. Brief in vitro restimulation identified GAD-reactive CD4^+^ T cells representing around 0.01% of blood T cells. The identification of multiple GAD-specific TCRs offers the opportunity to track these “sentinel” clonotypes longitudinally as markers of disease activity.

Antigen-specific T cell enrichment at sites of disease is well recognized in autoimmunity (26–28), including T1D where expanded T cells in the pancreas and pancreatic LNs are enriched for diabetes autoantigen specificity (29–31). Our data show that similar enrichment can be replicated in the skin following intradermal autoantigen injection, providing a minimally invasive means of monitoring disease-relevant responses. The frequency of GAD-reactive CD4^+^ T cells compares favorably with reports from pancreatic islets where 14 of 187 TCR clonotypes responded to preproinsulin peptides (22). The relative paucity of CD8^+^ GAD-reactive cells was unexpected given that 3%–40% of islet CD8^+^ T cells in T1D recognize proinsulin (31), but this may reflect donor HLA type or differences in CD4^+^ and CD8^+^ T cell trafficking. In earlier work using proinsulin-GNP conjugates we detected both GNP-reactive and proinsulin-reactive CD8^+^ T cells months after injection (18), suggesting kinetics may also be important.

A limitation of this study was that the controls did not include an injection of alum alone. However, in contrast to our previous study of intradermal proinsulin peptide conjugated to GNPs, where GNP-specific TCRs were found, we found no evidence of alum-specific TCR responses in the current dataset (18).

This approach offers several advantages over established methods such as ELISPOTs, HLA tetramers, and in vitro expansion assays (3, 7, 32). Unlike these techniques, scRNA-seq allows full transcriptional profiling of each antigen-specific T cell without preselecting functions or markers. It does not require prior knowledge of HLA restriction or defined epitopes and, through re-expression of paired TCRαβ chains in reporter lines, enables direct confirmation of antigen specificity independent of phenotype. This design also avoids the bias introduced by repeated in vitro stimulation, which can preferentially expand highly proliferative clones and distort their native phenotype.

Our data provide limited evidence for GAD-specific Treg responses under the conditions studied. Treg clusters in the LNs did not show prominent clonal expansion, and the TCR derived from a Treg population that we re-expressed did not demonstrate GAD reactivity. However, previous studies suggest that repeated antigen exposure may be required to induce or expand antigen-specific Tregs. For example, multiple rounds of GAD-alum administration have been reported to promote regulatory responses (33), and in the DIAGNODE-B pilot trial, increased IL-10 production in response to GAD was observed only after repeated dosing (34).

These findings raise the possibility that the single-dose challenge used here may preferentially recruit or reveal effector rather than regulatory responses. In this context, the approach described in our study could be applied longitudinally during antigen-specific immunotherapy to track the emergence and expansion of autoantigen-specific Tregs, as well as to monitor potential phenotypic transitions within clonally related T cell populations. While the current sampling time points aimed to recruit pre-existing, antigen-experienced T cells into the draining LNs, sampling at later time points may also enable assessment of de novo clonal expansion in response to autoantigen.

Across skin, LNs, and blood, GAD-reactive CD4^+^ T cells shared a conserved transcriptional program, dominated by NF-κB, RelA, STAT1, and STAT3 transcription factors and which are associated with Th17 and Th1 differentiation (35, 36). These cells expressed *CCR6* and *CXCR3* consistent with a Th17.1-like phenotype and the ability to migrate to islets. Interestingly, only GAD-reactive blood T cells expressed *IFNG* and *IL17A*, likely reflecting the timing of antigen exposure to GAD (recent in vitro restimulation versus earlier in vivo activation). We also identified *TXNIP* upregulation consistently across donors, in post-GAD LNs, aligning with reports of its elevation at T1D onset and modulation by verapamil (37).

While differential gene expression analysis at the single-cell level was limited by sample size, with few genes remaining significant after multiple testing correction, complementary pseudobulk and pathway analysis (TRRUST and GSEA) identified consistent transcriptional programs, increasing confidence in the biological interpretation of GAD-specific T cells (38, 39).

There has been considerable interest in public TCRα or TCRβ chains, or motifs within them that are enriched as people progress to T1D and in building repositories of known autoantigen-specific TCRs that can be used to track disease activity (40–45). In this context, it was interesting to note a shared GAD-specific TCRα between different donors differing by a single amino acid (B_post_GAD_LN-5, CAENGAGGTSYGKLTF; PBMC-1 [Donor D], CAENNAGGTSYGKLTF) and this TCRα (CAENNAGGTSYGKLTF) has been reported in the islets of a donor without T1D (31). Furthermore, a machine learning approach to peripheral blood TCR and BCR sequences has recently demonstrated success in classifying people with or without T1D (46). The repertoires enriched for autoantigen-specific TCRs that we have identified here may also contribute to a deeper understanding of shared autoantigen-specific TCRs. It would be particularly interesting to monitor these autoantigen-specific TCRs in T cell subsets such as Th17.1 and Treg cells over the course of immunotherapy, to better understand how autoantigen-specific cell phenotypes can be modulated at the level of individual clones. In addition, filtering of TCR sequences obtained from the LNs, blisters, and PBMCs for those with high homology to known autoantigen-specific TCRs offers a further pathway to enrich for autoantigen-specific TCRs and assess their associated cell phenotypes.

Despite these strengths, several caveats remain. The phenotypic heterogeneity in the phenotypes of GAD-specific T cells between donors and compartments may reflect differences in disease course or sampling limitations (47). In addition, the T cell clones detected peripherally may not perfectly represent those active in the pancreas, a limitation shared with all peripheral monitoring approaches. Nevertheless, the identification of *IFNG*^+^*IL17A*^+^*CSF2*^+^ CD4^+^ T cells in response to GAD is notable, as similar cells have been linked to accelerated C-peptide loss during alefacept therapy (48) and implicated in other autoimmune diseases (49–51). We recently reported that circulating Th17.1 cells coexpressing IL-2 and GM-CSF are targeted by ustekinumab and that reductions in this subset correlate with improved C-peptide preservation in T1D (20). This suggests that the GAD-reactive Th17.1-like cells identified here may have direct disease relevance.

GAD-reactive CD4^+^ T cells also expressed high levels of IL-32, previously associated with T1D progression (52–54) and IL-22, a Th17-associated cytokine elevated in human T1D (55, 56). Although IL-22 does not appear to directly drive β cell destruction in NOD mice (57, 58), it may contribute to tissue inflammation in human disease. We also observed nearly identical TRA sequences differing by only 1 amino acid among expanded GAD-reactive clonotypes from different donors. Public TCRs with such minimal junctional differences have been reported in T1D (40, 45), supporting convergent recognition of shared autoantigenic determinants. In the peripheral blood, we noted a switch from GAD-specific T cells with a Th17.1 *IL22*^+^ phenotype to a Th17.1 *IL2*^+^ phenotype, which may reflect the recent in vivo exposure to GAD-alum. It is hypothesized that multiple rounds of in vivo tolerogenic exposure to diabetes autoantigens are required to expand autoantigen-specific Treg populations (59, 60).

In summary, we describe 3 complementary methods for enriching and monitoring diabetes autoantigen-specific cells in humans. Sampling from the skin after intradermal antigen challenge enables isolation and molecular profiling of clonally expanded antigen-specific T cells, while LN aspiration and peripheral AIM assays provide complementary phenotypic data. Together, these methods provide a framework for longitudinal monitoring of antigen-specific T cells during immunotherapy and may accelerate the development of disease-modifying treatments for T1D.

## Methods

### Sex as a biological variable.

Our study examined male and female participants, and similar findings are reported for both sexes

### Study design.

After obtaining informed consent, 9 participants aged 18–50 years with T1D and positive for anti-GAD antibodies were recruited. Their demographic characteristics are shown in Supplemental Table 1. Six participants received single intradermal administration of 4 μg of GAD-alum in 100 μL (clinical grade, Diamyd Medical) into the deltoid region. Ultrasound-guided FNA of the axillary LN was performed by an expert radiologist before, and 2–7 days after GAD-alum administration in 5 participants. Out of 5 participants who received GAD-alum and had FNA, 3 also had a skin suction blister induced 5–13 days after the antigen administration, in order to sample lymphocytes from the site of injection. Skin blisters were harvested at the time of erythema and induration over the injection site as described below.

In addition, 3 participants who did not receive GAD-alum challenge had a suction blister taken from the unchallenged skin (i.e., no prior injection) of the deltoid region for the purpose of a negative control for the skin lymphocyte analysis (Supplemental Table 2).

### Blood samples.

PBMCs were isolated on Ficoll-Paque Plus (GE Healthcare Biosciences) gradient centrifugation from the peripheral blood of participants immediately prior to injection of GAD-alum. Samples were cryopreserved in CryoStor (Sigma-Aldrich) on the day of the collection and thawed on the day of the experiment. HLA typing was performed by the Anthony Nolan Trust (Supplemental Table 3).

### Suction blisters.

Skin suction blisters were raised as described previously (18, 61). Briefly, suction cups were applied to the deltoid region of participants’ arms, at the site of previous injection. Skin suction blisters were performed by gradually applying negative pressure (up to 60 kPa) from a suction pump machine VP25 (Eschmann) through a suction blister cup with a 15-mm hole in the base (UHBristol NHS Foundation Trust Medical Engineering Department) for 2–4 hours until a unilocular blister had formed within the cup. The cup was left in place for a further 30–60 minutes to encourage migration of lymphocytes into the blister fluid. The cup was then removed, and the blister fluid aspirated using a needle and syringe. Blister fluid was immediately suspended in 10 mL heparinized RPMI (Gibco) with 5% fetal calf serum (FCS). Cells were counted then washed once and resuspended in an appropriate volume for scRNA-seq. Samples were used fresh on the day of the collection.

### Ultrasound-guided FNA of LNs.

Ultrasound-guided FNA was performed as previously reported (17). Under aseptic conditions, the skin and s.c. tissues down to the identified LN were infiltrated by 1–2 mL of 1% (w/v) xylocaine with 1:200,000 adrenaline. Under real-time ultrasound visualization, using a 21-gauge needle and a 5-mL syringe, the LN cortex was sampled. One to 2 passages were used per sample. The LN sample was immediately transferred to tissue culture medium (5% [v/v] FCS [BioSera] in RPMI 1640 medium [Gibco]) and transported to the laboratory at ambient temperature. PBMCs were isolated using Lymphoprep (StemCell Technologies) and frozen in Cryostor CS10 (Sigma-Aldrich) until required.

### In vitro stimulation and flow cytometric sorting of CD4^+^ T cells.

The AIM assay was performed similarly to that previously published (7). Briefly, PBMCs from donors before in vivo injection of GAD-alum were thawed and stimulated for 18 hours in 1 of 4 conditions in X-VIVO media with 10% human AB serum (Merck) at 4 × 10^6^ cells/mL: (a) unstimulated; (b) whole GAD (Diamyd Medical) (no alum) in buffer (final concentration 10 μg/mL); (c) control, using the GAD dilution buffer only; and (d) SEB injection (final concentration 50 ng/mL). Following this, cells were stained with a cocktail of flow cytometry, cell hashing, and CITE-seq antibodies (Supplemental Table 34). CD4^+^ T cells were then sorted as follows (Supplemental Figures 15 and 16): for SEB, GAD, and buffer control — effector T cells were defined as CD154^+^ and either CD69^+^ or CD137^+^ or both; Tregs were defined as CD154^–^CD137^+^ and either CD69^+^ or GARP^+^ or both. For GAD and buffer control conditions, all cells were sorted from the positive gate. For SEB-stimulated, 100 activated Tregs and 1000 activated effectors were sorted. From the unstimulated condition, 1000 cells negative for all 4 activation markers were sorted. All cells were processed for scRNA-seq on the day of sorting.

### scRNA-seq.

LN and injected blister samples were processed using a 10x Genomics V1 human 5′ GEX kit and libraries were constructed following standard 10x Genomics protocols. Samples were pooled and sequenced on Illumina NextSeq and MiSeq. Demultiplexed samples were processed individually using the 10x Genomics CellRanger 3.0.2 pipeline and GRCh38-1.2.0 with vdj GrCh38-alts-ensembl 2.0.0. For control blisters and PBMCs, the 10x Genomics V2 human 5′ GEX kit and CellRanger 6.1.1 pipeline were used. Data were further processed using the Seurat V4 package in R (62) and DoubletFinder (63) and manual gating were used to remove doublets. TCR sequences and clone numbers were added to the Seurat object metadata slot. TCR clone numbers refer to data generated in the CellRanger pipeline, even if clones of the same cell did not meet gene expression QC thresholds. TCRs were compared at the amino acid level. To be defined as a clone, CDR3 sequences were required to be identical as were V and J gene usage. Amino acid changes outside of the CDR3 were permissible. Morisita-Horn indexes were calculated from the Seurat objects after QC. Raw data contained red blood cells, but these were removed automatically in the QC process due to low levels of UMIs and low unique genes identified. Cell clusters were determined by Seurat and annotations were manually assigned using curated lists of genes. We performed batch correction with Harmony (64) to produce a single Seurat object. Data were further analyzed in R using scPubr, ggplot2, cowplot, ggpubr, and tidyverse packages. Differentially expressed genes (DEGs) were identified in Seurat and TRRUST pathways were analyzed in Metascape v3.5.20240901. Pseudobulking was also performed in Seurat. Total cell numbers per donor and in each cluster and TCR frequencies by donor after QC are shown in Supplemental Tables 30–32.

### Antigen specificity experiments.

TCR specificity was determined as previously described (8, 18). Briefly α-chain and β-chain genes connected by the porcine teschovirus-1 2A peptide gene were engineered into a murine stem cell virus–based retroviral vector. T cell transductants were generated by retroviral transduction of the 5KC murine T cell hybridoma cells lacking endogenous TCR expression (8, 18), expressing fluorescent proteins downstream of the NFAT promoter and expressing human CD4 or CD8 co-receptors according to the original T cell phenotype. 5KC cells were spin-infected with replication-incompetent retroviruses that were produced from Phoenix cells (ATCC) transfected with the retroviral vectors and the pCL-Eco packaging vector. Cells transduced with retroviruses were then selected by sorting for CD3 expression (CD3ε MicroBead Kit, mouse, Miltenyi Biotec).

A codon-optimized *GAD2* gene (GAD/GAD65 protein) was cloned into the third-generation lentiviral transfer vector backbone pELNS (provided by James Riley, University of Pennsylvania, Philadelphia, Pennsylvania, USA) containing a rat CD2 (rCD2) marker gene preceded by a P2A self-cleaving sequence: XbaI-Kozak-*GAD2*-XhoI-P2A-rCD2-SalI-Stop. Lentiviral particles were produced using HEK293T cells (ATCC) and envelope plasmid pMD2.G (gift from Didier Trono, Addgene plasmid 12259), and packaging plasmids pMDLg/pRRE (gift from Didier Trono, Addgene plasmid 12251) and pRSV-Rev (gift from Didier Trono, Addgene plasmid 12253) and used to insert GAD into participants’ EBV-transformed B cells (LCLs).

5KC cells bearing TCRs were incubated for 48 hours with autologous LCLs with antigens as indicated at 1 × 10^6^ 5KC/mL and 1 × 10^6^ LCLs/mL in 200 μL RPMI 1640 (Gibco) with penicillin/streptomycin (Gibco), 10% FCS (BioSera), and 2-mercaptoethanol (Thermo Fisher Scientific) in 96-well round-bottom plates. The positive control was 5 μL anti-CD3/CD28 microbeads (Dynabeads). Flow cytometry was performed on a BD FACSCanto II. Flow cytometry data were analyzed using FlowJo 10.7.1. Murine IL-2 ELISAs were performed on undiluted cell culture media following the manufacturer’s instructions (BioLegend).

### Statistics.

DEGs that defined cell clusters in Seurat were determined using Seurat’s inbuilt statistical methods using the Wilcoxon rank-sum test, using either only.pos=TRUE as described in the results section. DEG pathway analysis was conducted using Metascape standard parameters (65) on all DEGs with an uncorrected *P* value of less than 0.05 (*P* < 0.5 for pseudobulk). Metascape parameters using Benjamini-Hochberg *P* value correction are reported. TCR-5KC reactivity to GAD-alum presented by EBV-transformed autologous B cells (LCLs), or GAD-expressing LCLs was determined using a 1-tailed *t* test compared to TCR-5KC with LCLs alone. A *P* value of less than 0.05 was considered significant. Error bars on graphs represent SD.

### Study approval.

This study was carried out with the approval of the UK Research Ethics Service (Wales Research Ethics Committee 1, reference number 13/WA/0113). Written informed consent was obtained from all participants. The study was conducted in compliance with the principles of the Declaration of Helsinki (1996) and the principles of Good Clinical Practice and in accordance with all applicable regulatory requirements, including but not limited to the Research Governance Framework and the Medicines for Human Use (Clinical Trial) Regulations 2004, as amended in 2006. Written informed consent was received for the use of photographs and the record of informed consent has been retained.

### Data availability.

The scRNA-seq data have been deposited in ArrayExpress (https://www.ebi.ac.uk/biostudies/arrayexpress/studies/E-MTAB-17069). The Supporting Data Values file contains the data for graphs in the figures.

## Author contributions

SJH conceptualized the study, curated data, performed formal analysis, acquired funding, conducted investigations, developed methodology, created visualizations, wrote the original draft, and reviewed and edited the manuscript. EJSR, TCT, and EW conducted investigations, developed methodology, and reviewed and edited the manuscript. MN, LL, GD, AKS, and AL contributed methodology and resources and reviewed and edited the manuscript. RA curated data, developed software, and reviewed and edited the manuscript. JD and AH conducted investigations, administered the project, and reviewed and edited the manuscript. JAP acquired funding, contributed methodology, supervised the study, and reviewed and edited the manuscript. PN and DW contributed methodology and reviewed and edited the manuscript. PY and MH conducted investigations, developed methodology, and reviewed and edited the manuscript. FSW supervised the study and reviewed and edited the manuscript. TIMT conducted investigations, developed methodology, contributed resources, and reviewed and edited the manuscript. CMD conceptualized the study, acquired funding, supervised the study, and reviewed and edited the manuscript. DT conceptualized the study, acquired funding, and reviewed and edited the manuscript.

## Conflict of interest

AL is an employee of Diamyd Medical AB. CMD is involved as a paid advisor to Provention Bio and Sanofi and holds a patent (WO2016162495) jointly with Biodexa plc for GNPs. DW holds patents for peptides, tolerization-inducing composition, FVIII peptides and their use in tolerizing hemophiliacs, composition, disease markers, tolerogenic peptides from myelin basic protein, peptide selection method, AIM assay, tolerogenic peptides, T cell activation, and improvements relating to influenza vaccine (US8961986B2, US9775880B2, US20180327463A1, US10377800B2, US20050058643A1, US10646589B2, US20150071967A1, US20190275126A1, US20190330298A1, AU2014200237B2, US20190300573A1, US20200046802A1, WO2024023521A1, WO2024023522A1, and US20260034200A1). TT reports consultancy income from SAB Bio, research income from GlaxoSmithKline and Galvani Bioelectronics, and patent numbers WO2021176044 A1 and WO2024121173A1.

## Funding support

The following organizations provided funding support:

- Diabetes Research and Wellness Foundation Professor David Matthews Research Fellowship to SJH.
- The “Steve Morgan Foundation Type 1 Diabetes Grand Challenge” by Breakthrough T1D UK (formerly JDRF) and SMF (grant numbers 2-SRA-2024-1474-M-N and 2-SRA-2024-1473-M-N) to JAP and DT.

## Supplementary Material

Supplemental data

Supplemental tables 1-34

Supporting data values

## Figures and Tables

**Figure 1:**
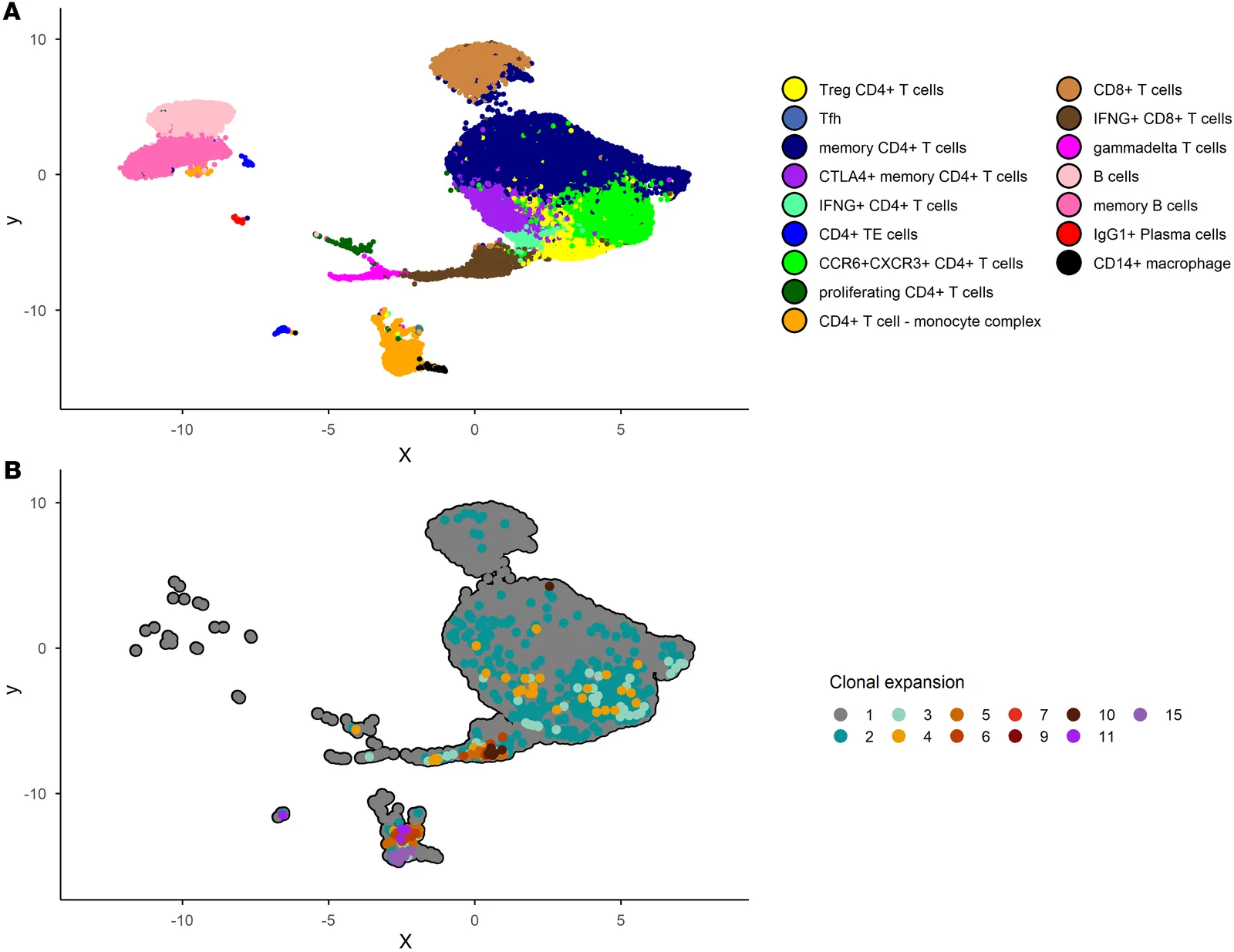
scRNA-seq of lymph node and skin blister samples following intradermal GAD-alum injection. (**A**) UMAP plot showing all pre- and post-GAD lymph node (LN) cells and post-GAD blister cells, clustered in Seurat and colored by cell type. (**B**) UMAP plot of T cells expressing paired TCRs, colored by clonal expansion number. Data are pooled from 5 donors. TCRs were compared at the amino acid level. To be defined as a clone, CDR3 sequences were identical, as were V and J gene usage. Amino acid changes outside of the CDR3 were permissible. Morisita-Horn indexes were calculated from the Seurat objects after QC. Total cells in this analysis were 67,780 LN cells and 2620 blister cells.

**Figure 2:**
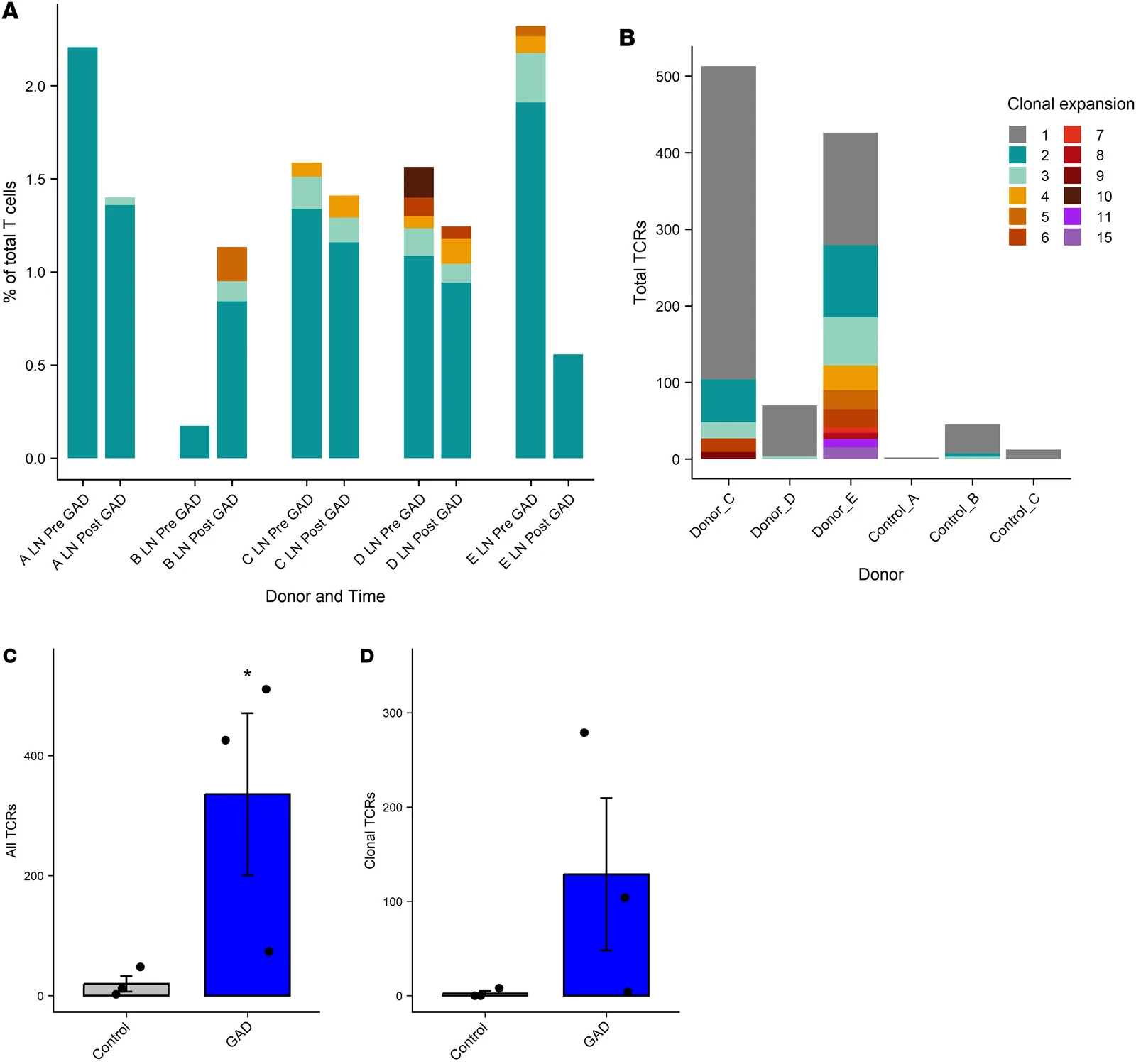
TCRs in LN and skin blister samples following intradermal GAD-alum injection. (**A**) Bar graph showing the proportion of clonally expanded T cells in the LNs, expressed as a percentage of all T cells with sequenced TCRs and colored by expansion size. (**B**) Total number of TCRs sequenced from each of the skin blisters, colored by clonal expansion number. Clonal expansion legend colors refer to **A** and **B**. (**C**) Total number of TCRs in blisters. (**D**) Number of clonal TCRs in blisters. **C** and **D** show pooled data from 3 donors in each group. *P < 0.05 by 1-tailed *t* test. TCRs were compared at the amino acid level. To be defined as a clone, CDR3 sequences were required to be identical, as were V and J gene usage. Amino acid changes outside of the CDR3 were permissible. Morisita-Horn indexes were calculated from the Seurat objects after QC. A total of 52,959 TCRs were analyzed across all samples.

**Figure 3:**
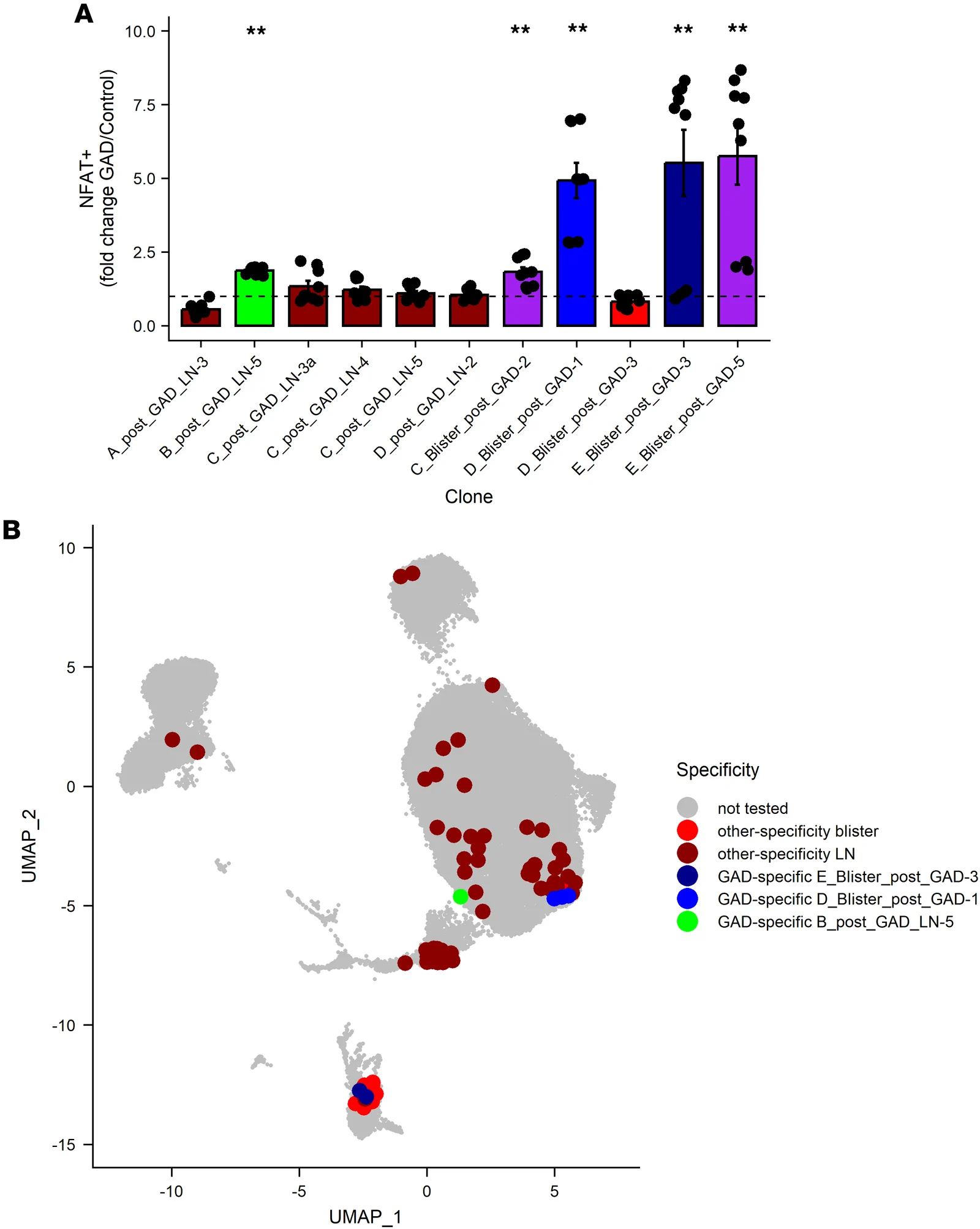
GAD specificity of re-expressed TCRs. (**A**) TCRs identified by scRNA-seq were re-expressed in 5KC reporter cells. TCRs showing positive responses when screened with GAD-alum were retested using autologous GAD-expressing LCL lines. **P < 0.01 by 1-tailed *t* test. Data are pooled from duplicate observations in 3 independent experiments. (**B**) UMAP plot showing the distribution of all re-expressed TCRs mapped back onto the single-cell dataset, illustrating phenotypes of GAD-specific T cells compared with those of other specificities, colored by clone ID. Data are pooled from 5 donors. A total of 38 TCR sequences were analyzed (Tables 1 and 2).

**Figure 4:**
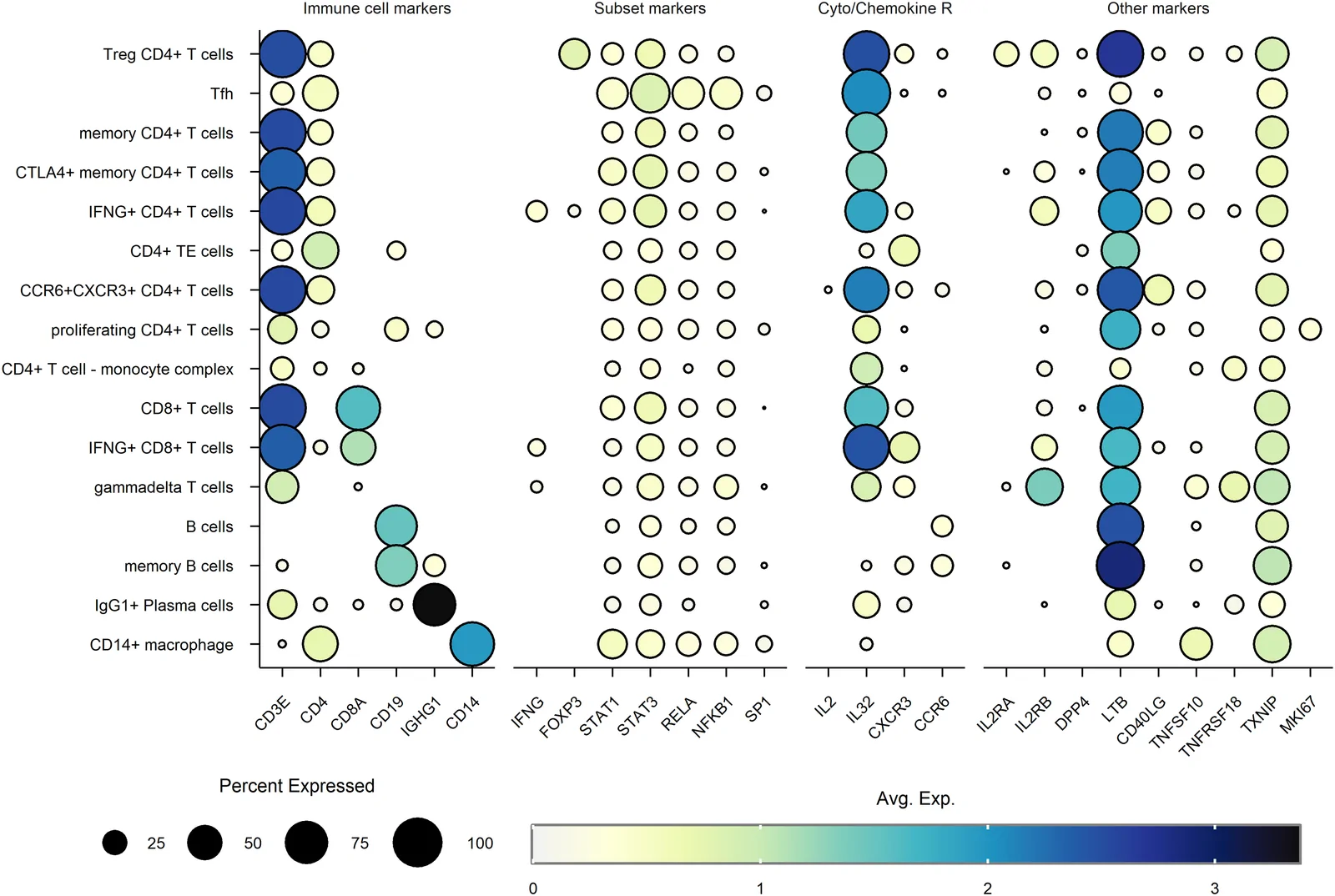
Gene expression profiles of cell subsets. Dot plot showing expression of major subset markers across scRNA-seq clusters from combined LNs and blister samples. Data are pooled from 5 donors. Total cells in this analysis were 67,780 LN cells and 2620 blister cells.

**Figure 5:**
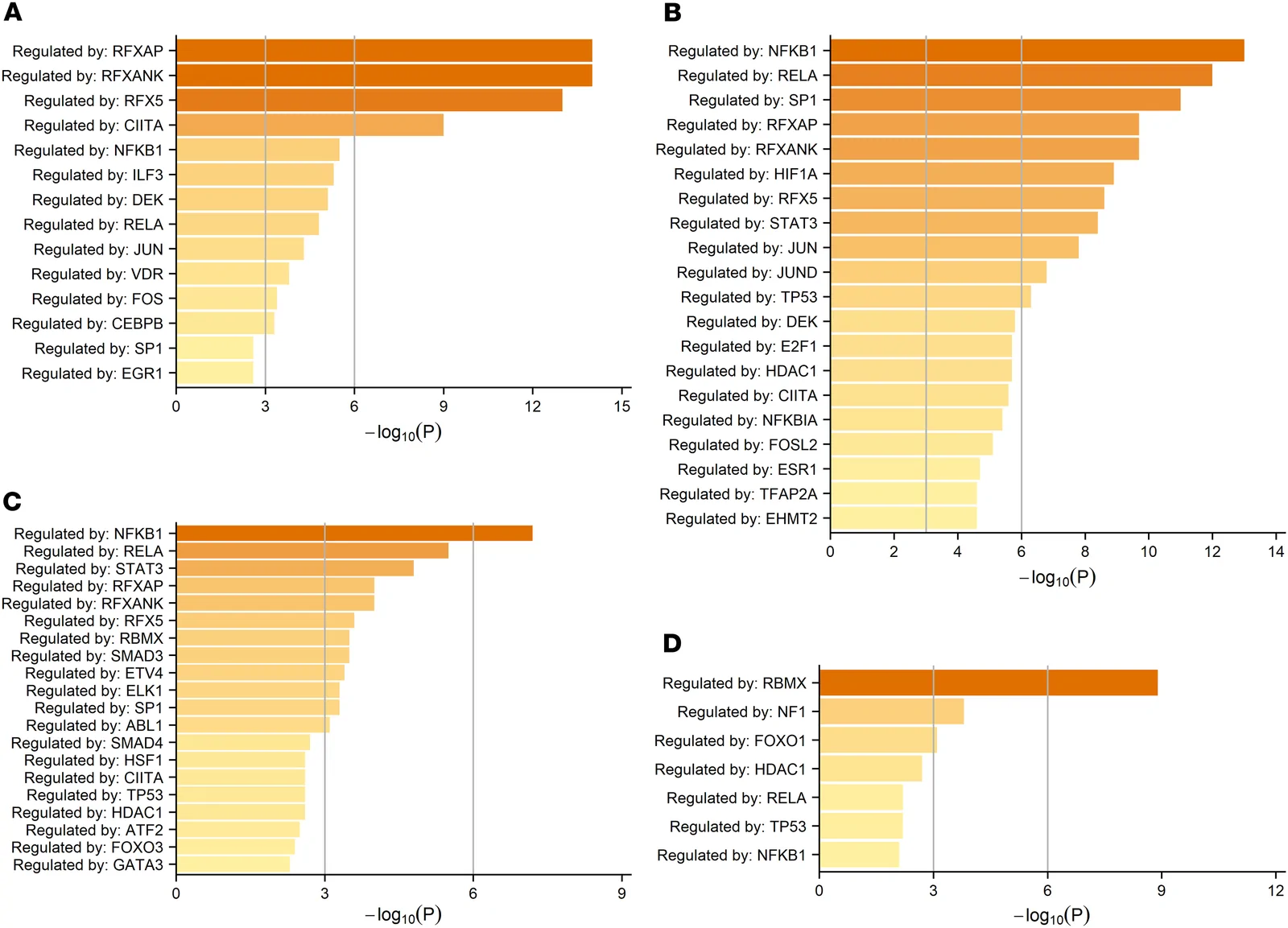
Gene expression profiles of T cells following in vivo GAD. (**A**) Transcription factors identified by TRRUST analysis of all genes upregulated in blister T cells following GAD injection compared with control blisters. (**B**) Transcription factors identified by TRRUST analysis of genes upregulated in conventional CD4^+^ T cells from blisters compared with pre-GAD LN samples. (**C**) Transcription factors identified by TRRUST analysis of all upregulated genes in clonally expanded CD4^+^ T cells post-GAD vs. pre-GAD in the LNs. (**D**) Transcription factors identified by TRRUST analysis of all genes upregulated in clonally expanded CD8^+^ T cells post-GAD vs. pre-GAD in the LNs. Data are pooled from 5 donors in LN groups and 2 donors in blister groups. A total of 740 clonally expanded T cells were analyzed. DEG pathway analysis was conducted using Metascape standard parameters on all DEGs with an uncorrected *P* value of less than 0.05 and Metascape parameters using Benjamini-Hochberg *P* value correction are reported.

**Figure 6:**
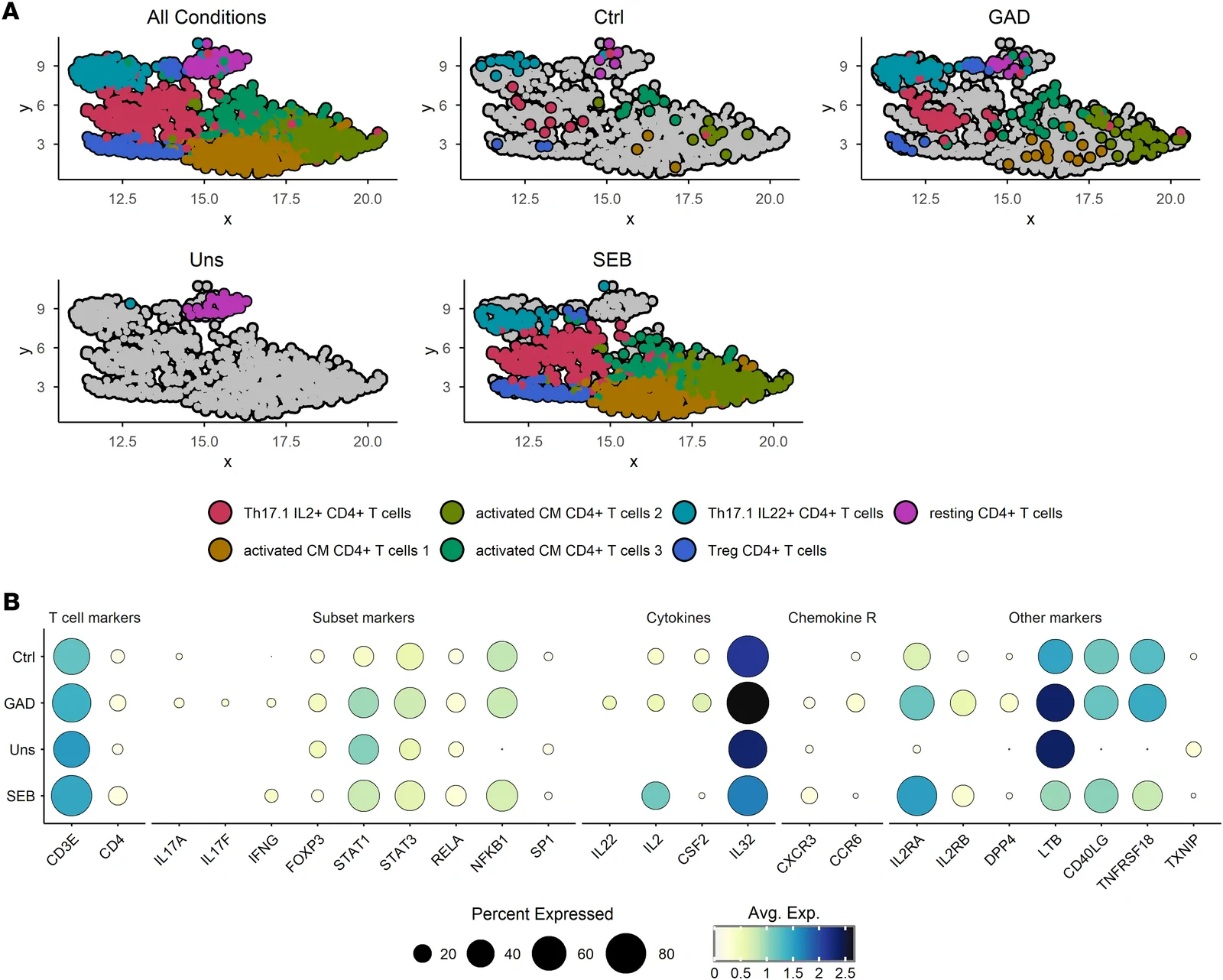
In vitro stimulation of CD4^+^ T cells from peripheral blood. (**A**) UMAP plots of all flow-sorted cells separated by in vitro restimulation condition. (**B**) Dot plots showing expression of key markers across each cluster. Data are pooled from 6 donors and 3486 total cells.

**Figure 7:**
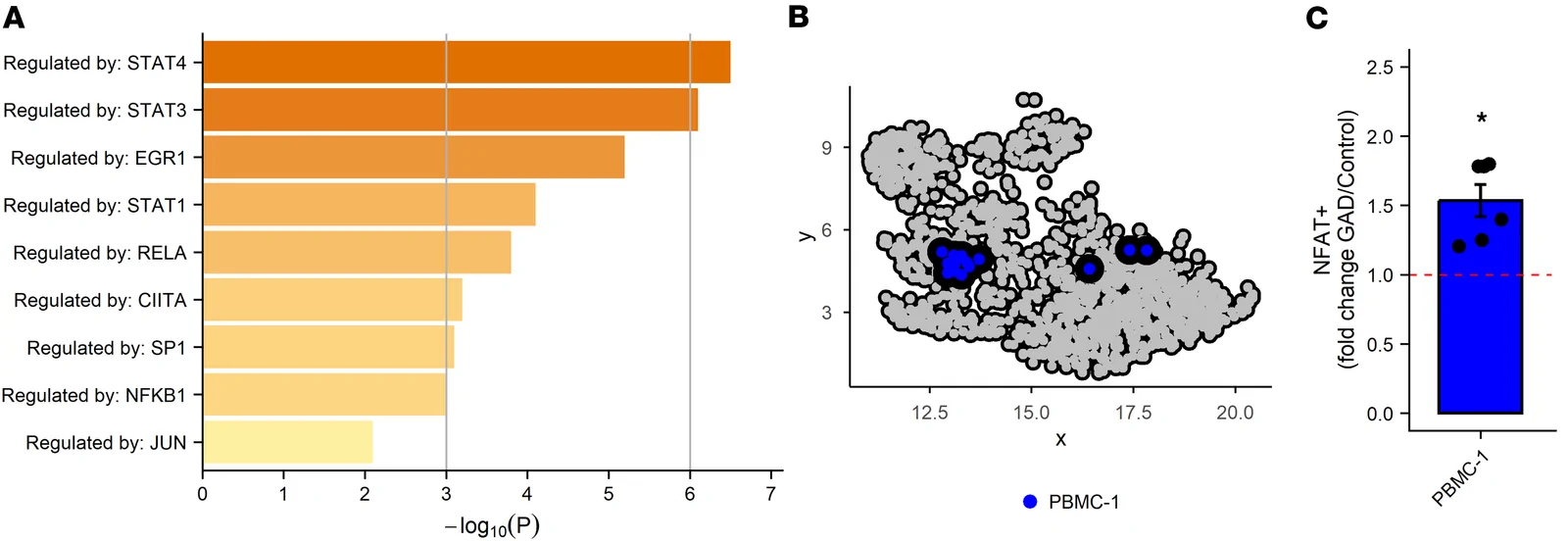
GAD-reactive T cells from peripheral blood have Th17.1 phenotypes. (**A**) Transcription factors identified by TRRUST analysis of genes upregulated in GAD-activated compared with control-activated cells. (**B**) UMAP plot highlighting a clonally expanded population of cells (“PBMC-1”) detected only in the GAD-activated sample. Data are pooled from 6 donors. (**C**) PBMC-1 TCR re-expressed in 5KC reporter cells and stimulated with control or GAD-expressing LCLs. *P < 0.05 by unpaired 1-tailed *t* test from 3 independent experiments. A total of 693 T cells from Ctrl and GAD-stimulated conditions were analyzed in this figure. DEG pathway analysis was conducted using Metascape standard parameters on all DEGs with an uncorrected *P* value of less than 0.05 and Metascape parameters using Benjamini-Hochberg *P* value correction are reported.

**Table 2:**
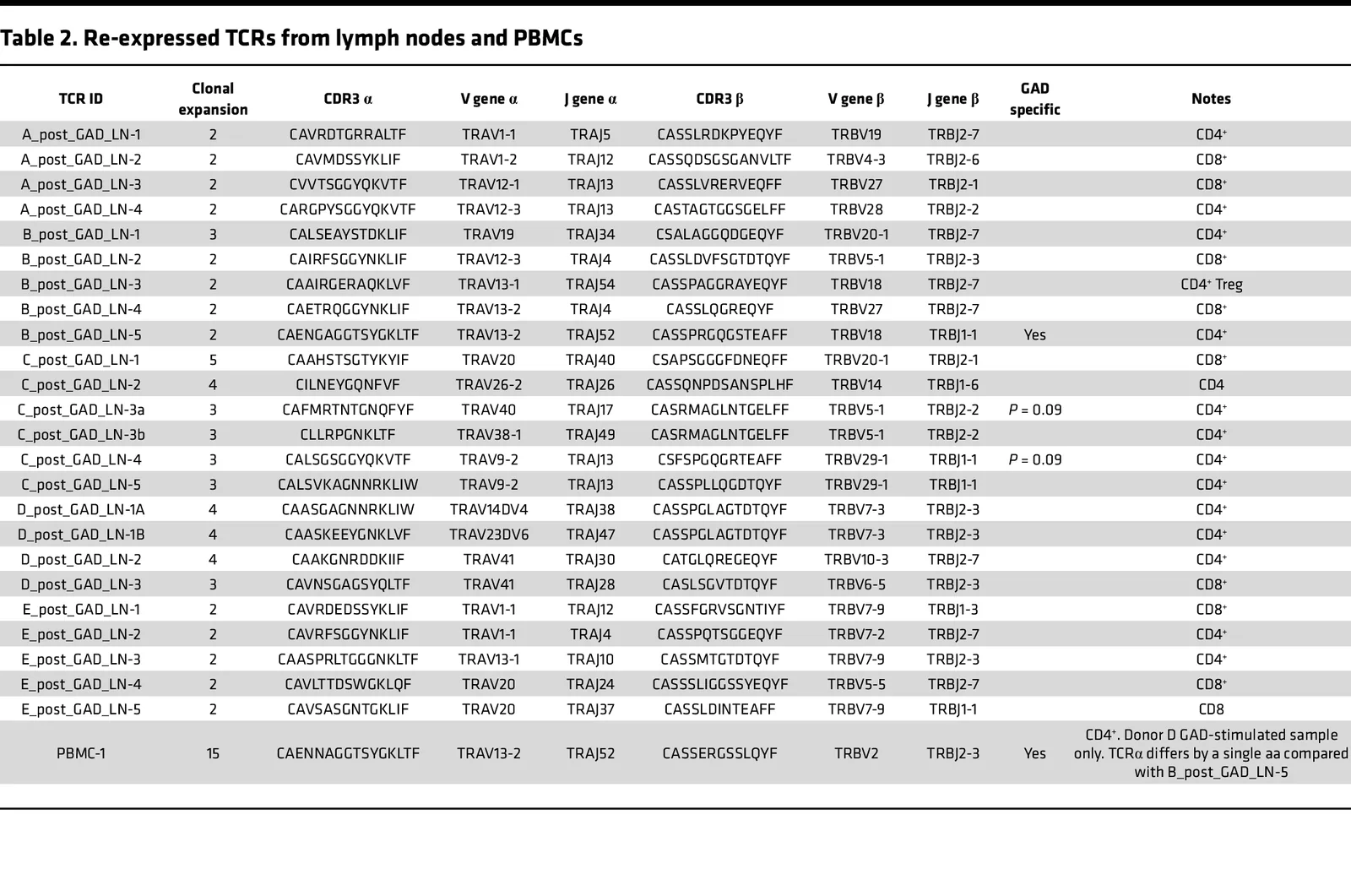
Re-expressed TCRs from lymph nodes and PBMCs

**Table 1:**
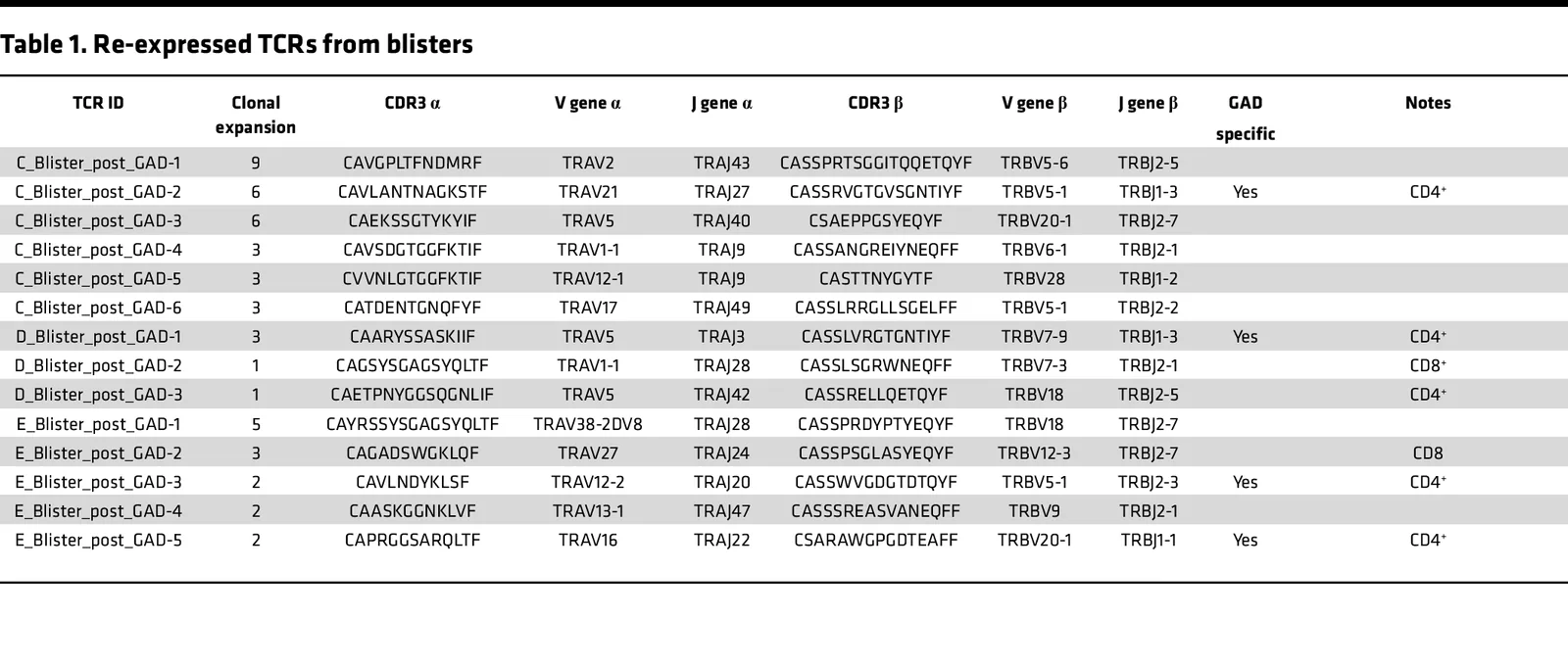
Re-expressed TCRs from blisters

## Version 1 09/01/2026

In-Press Preview

## Version 2 10/22/2026

Electronic publication
