# Supplementary material for: Tracking GAD-specific T cell expansions in type 1 diabetes by intradermal GAD-alum challenge
Source: JCI Insight. 2026 Sep 1;11(20):e203119. doi: 10.1172/jci.insight.203119 (PMC13726560; doi:10.1172/jci.insight.203119)
Supplement: Supplementary file 1 [file jciinsight-11-203119-s238.pdf]

Supplemental figures

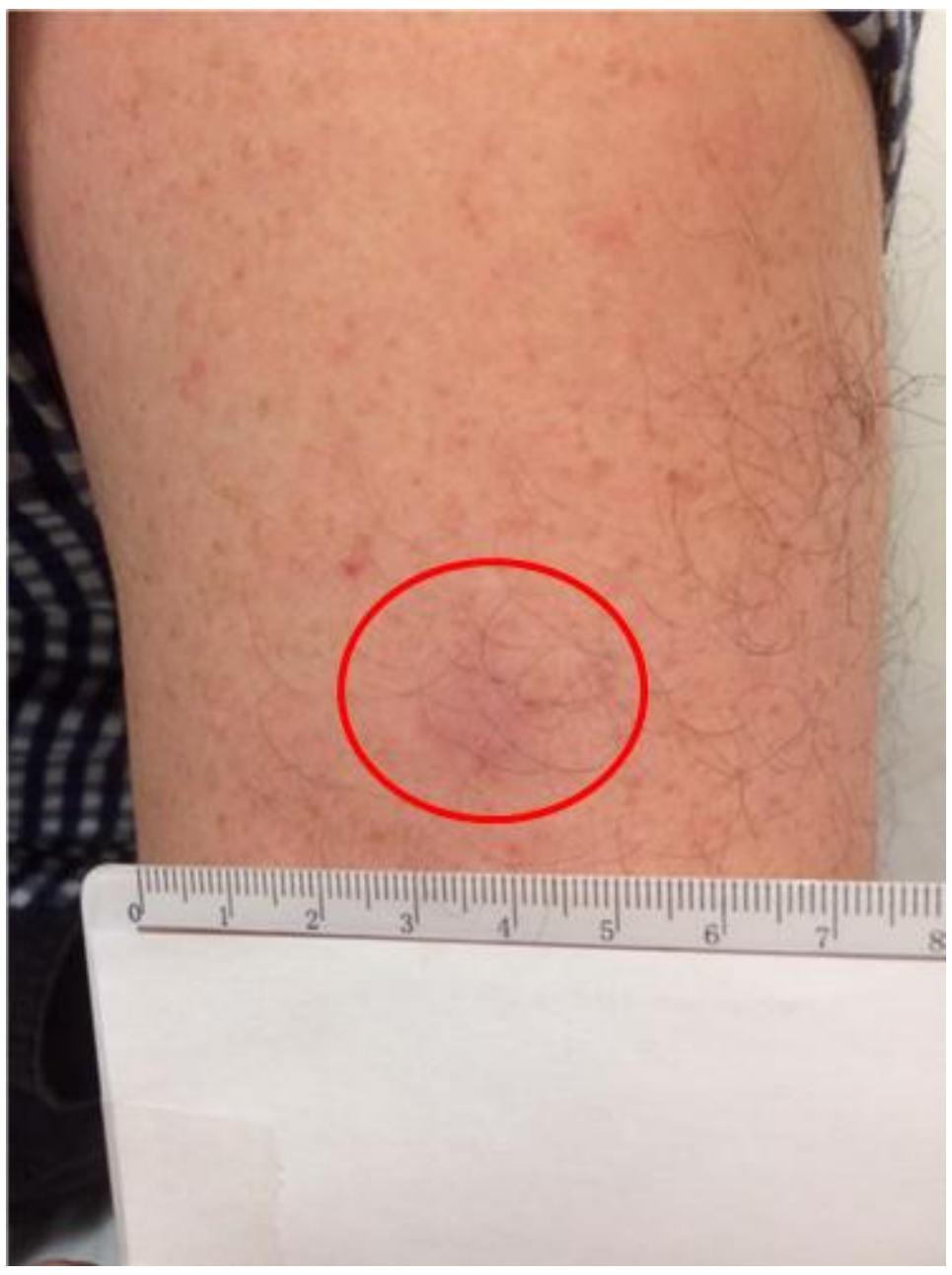

Supplemental Figure 1. Example of delayed skin reaction following intradermal GAD-Alum injection.

Representative clinical photograph showing the characteristic delayed erythematous induration (2–3 cm diameter) that developed at the site of intradermal GAD-Alum injection. The reaction typically appeared 2–3 days post-injection and resolved spontaneously within 15 days.

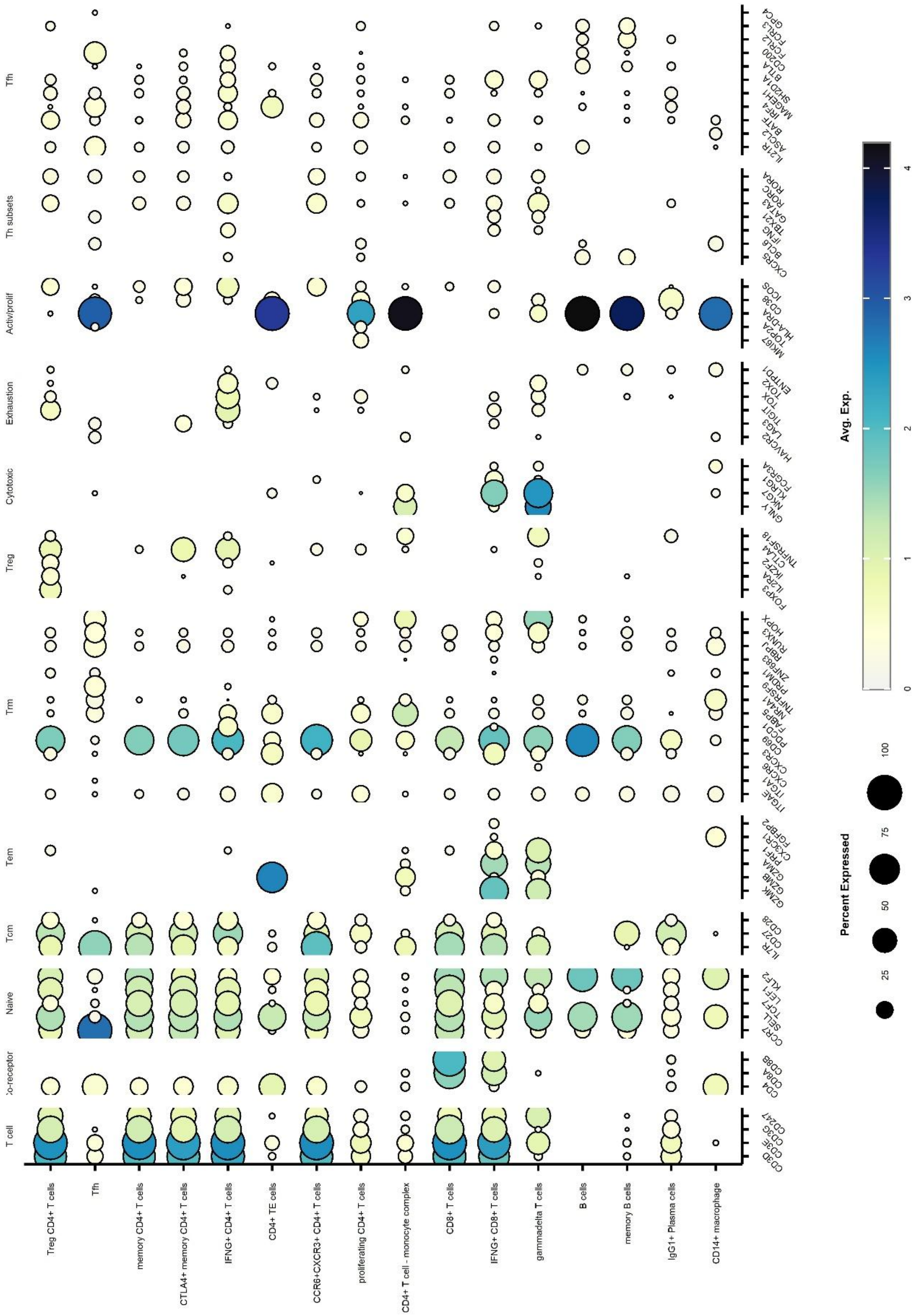

Supplemental Figure 2: DotPlot of canonical T cell subset Markers in LN and Blister cell clusters. Data are pooled from five donors' LNs and two donors' blisters

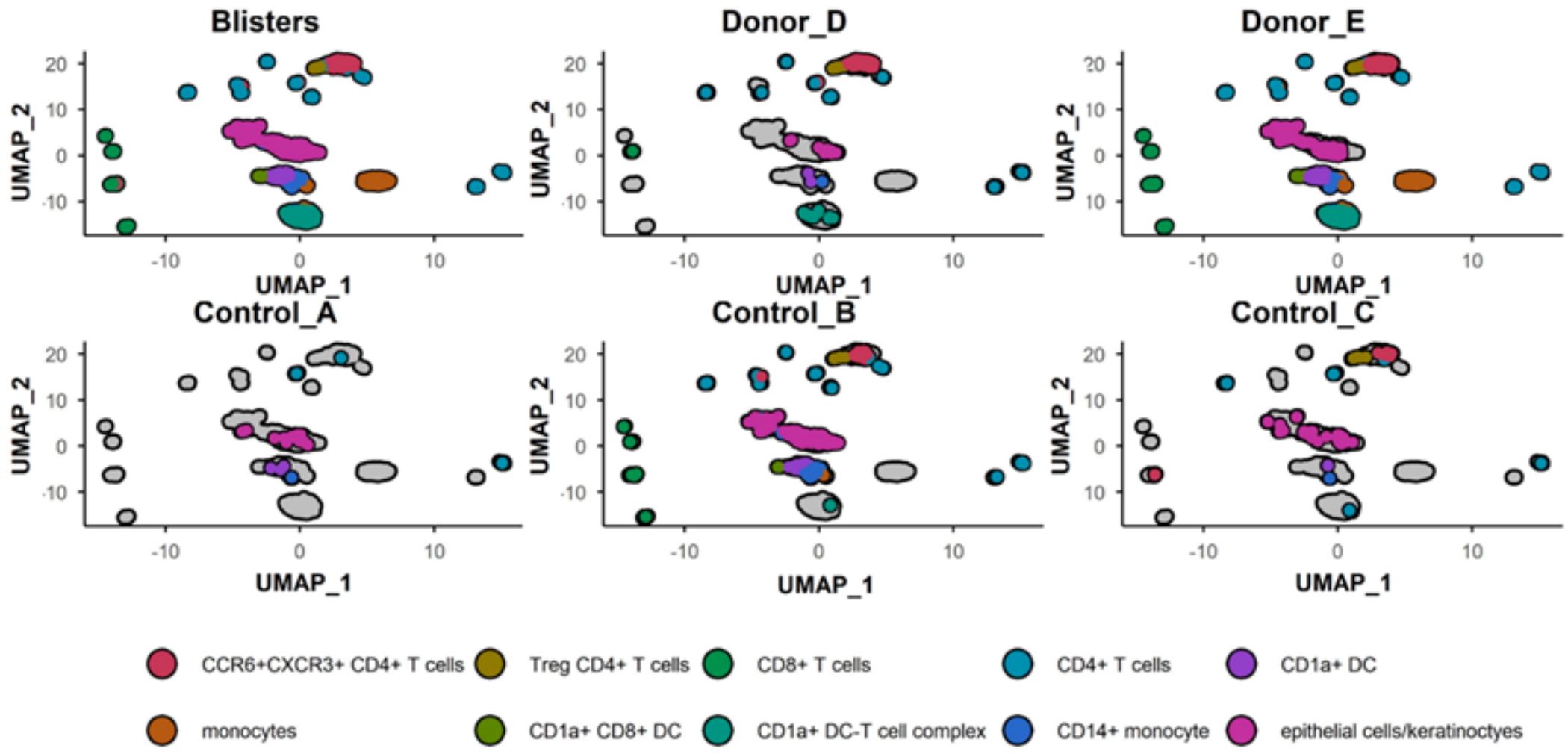

Supplemental Figure 3. Single-cell RNA sequencing of cells from GAD-injected and control blisters. UMAP plots of all cells from GAD-injected and control blisters, clustered in Seurat and coloured by cell type. Data are shown both combined and separated by donor.

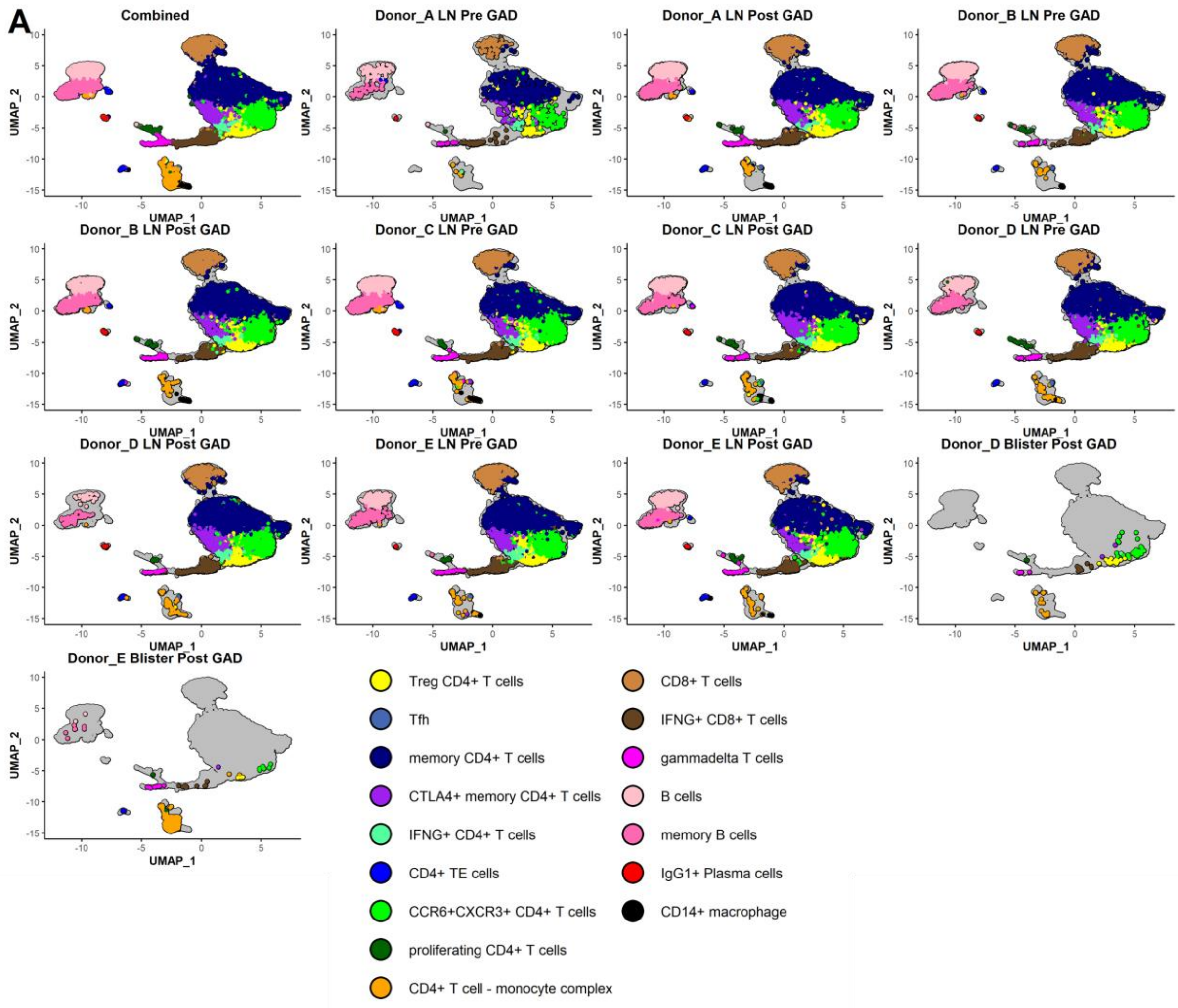

Supplemental Figure 4. Single-cell RNA sequencing of cells from GAD-injected blister and pre- and post-GAD lymph nodes.

UMAP plots of all cells from GAD-injected blisters and lymph node samples collected before and after GAD injection. Cells were clustered in Seurat and coloured by cluster identity. Combined data are shown, followed by plots split by donor and sampling timepoint.

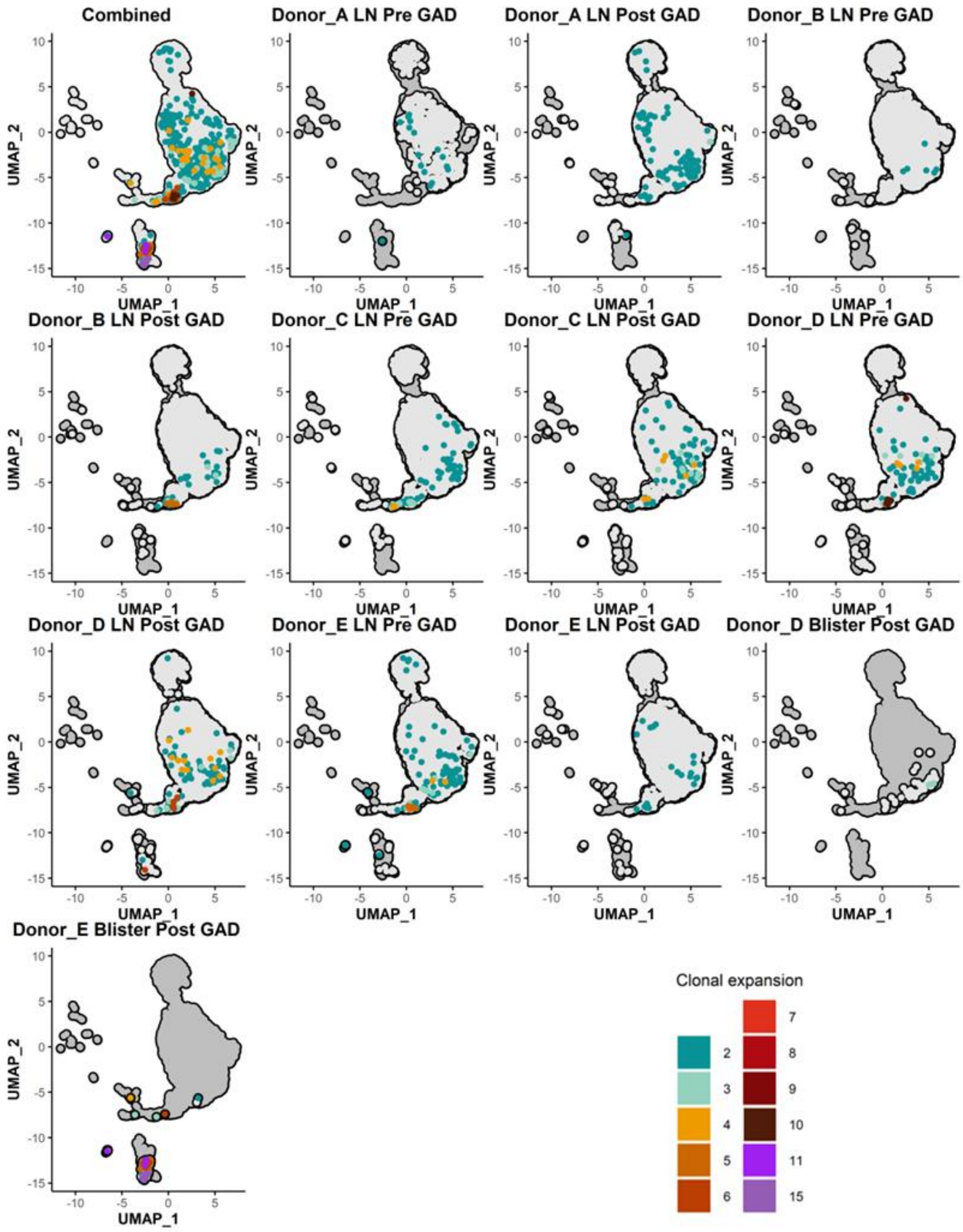

Supplemental Figure 5. Clonal expansions of TCRs in lymph nodes and blisters. UMAP plots of all cells from lymph node and blister samples, clustered in Seurat. Combined data are shown, followed by plots split by donor and sampling timepoint. Cells are coloured according to TCR clonal expansion.

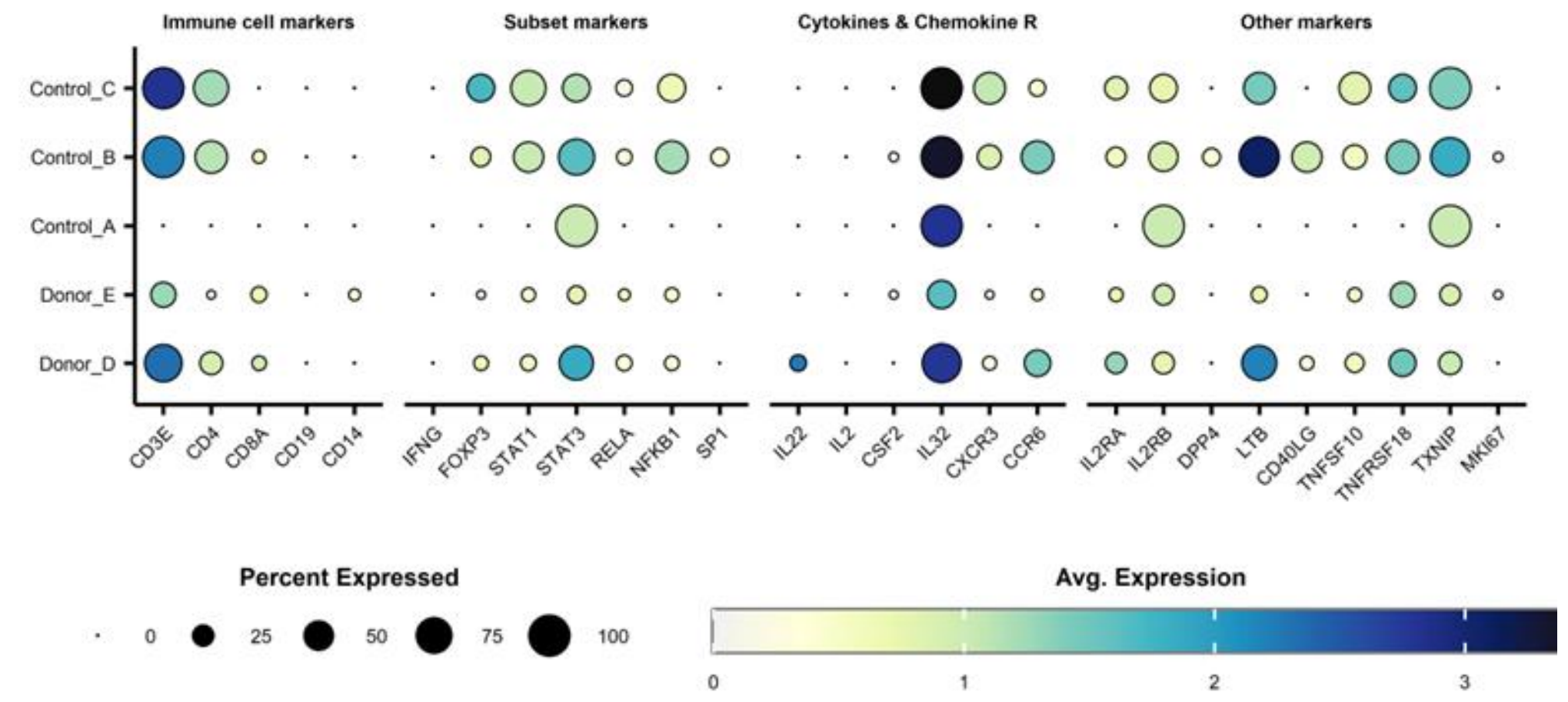

Supplemental Figure 6. Comparison of gene expression in CD4+ T cells from blisters.

Gene expression profiles of CD4+ T cells from blister samples, shown separately for each donor to illustrate inter-individual variation in transcriptional signatures.

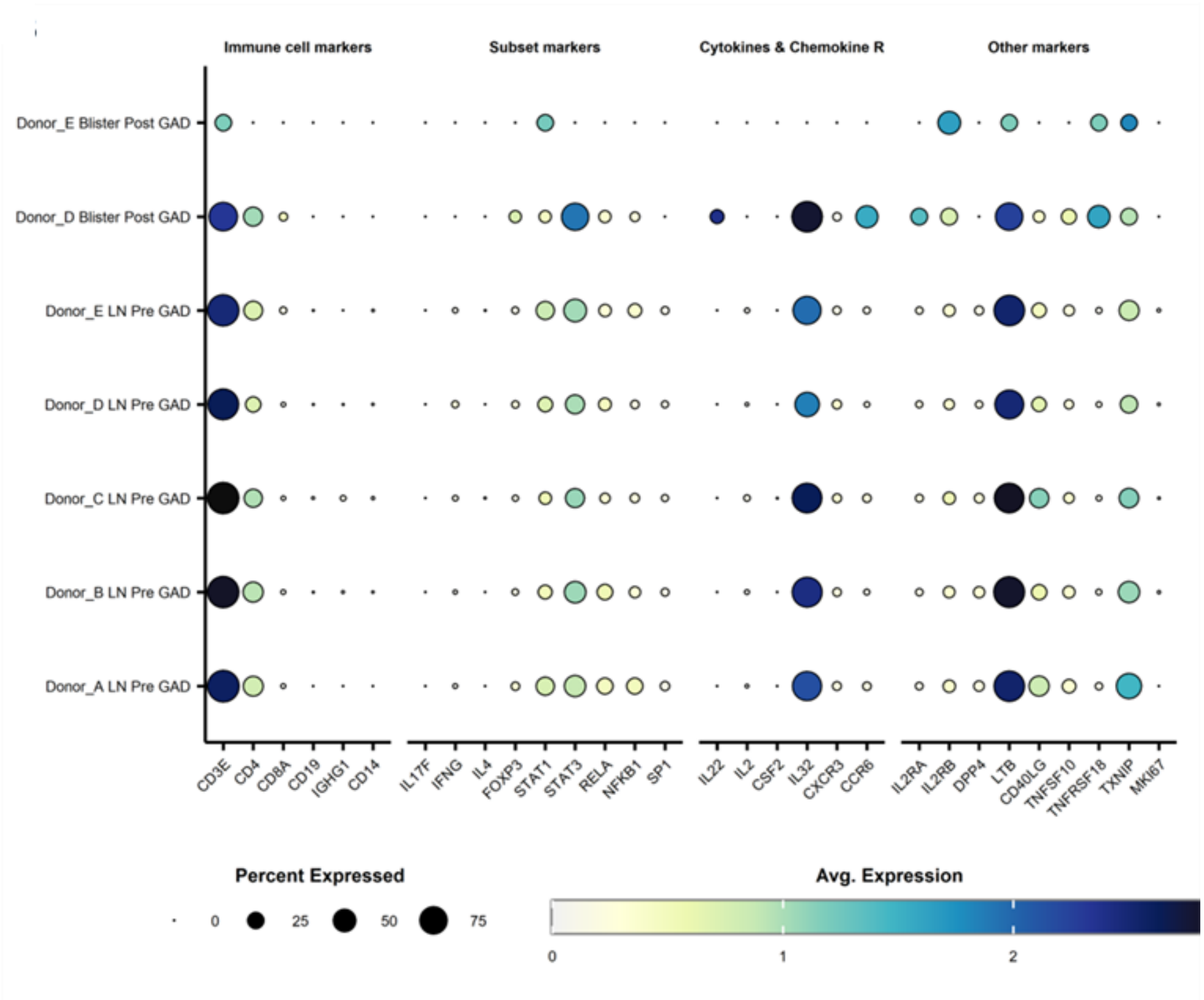

Supplemental Figure 7. Comparison of gene expression in CD4+ T cells from blisters versus pre-GAD lymph nodes.

Differential gene expression profiles of CD4+ T cells from GAD-injected blister samples compared with pre-GAD lymph nodes, shown separately for each donor.

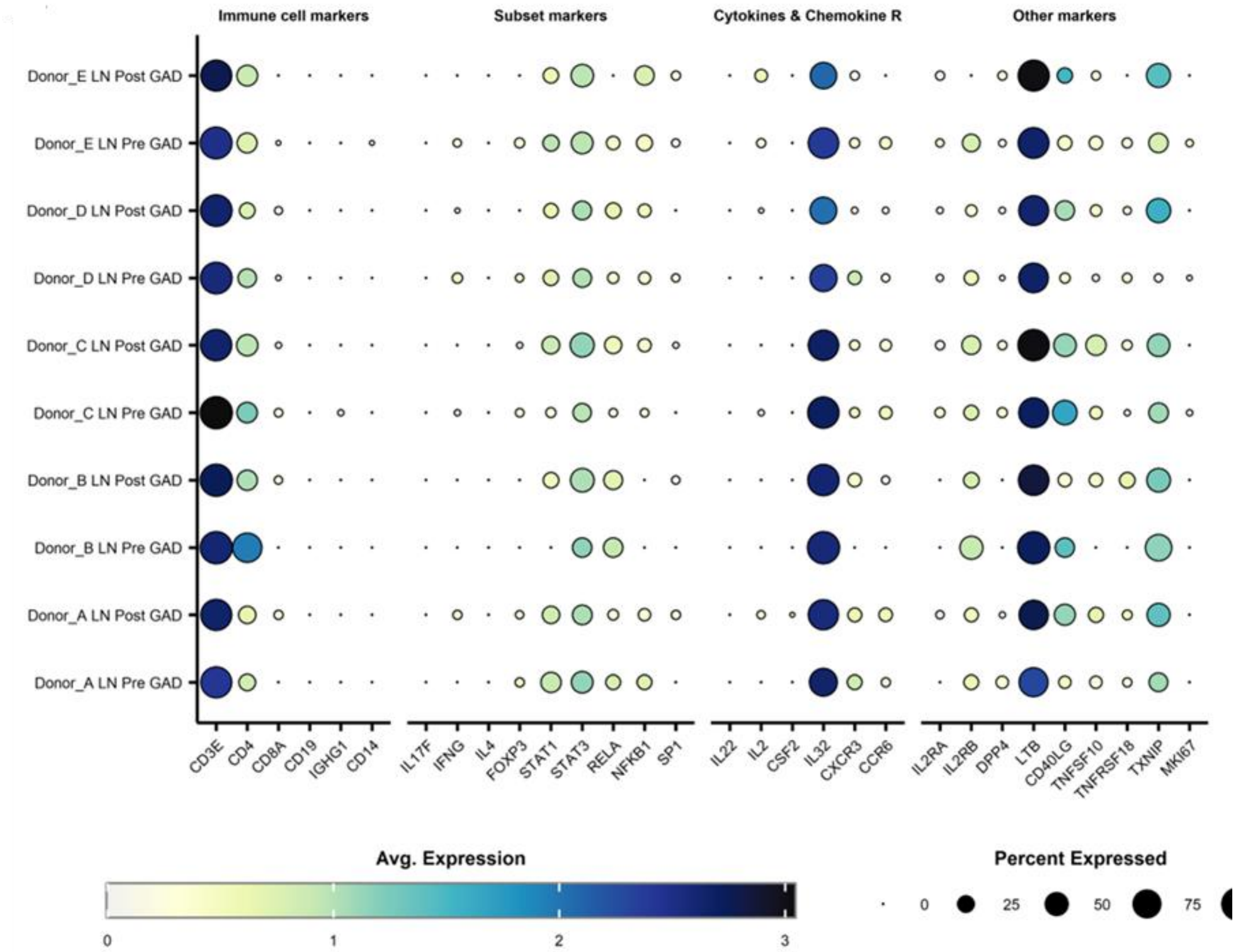

Supplemental Figure 8. Gene expression in clonally expanded CD4+ T cells in lymph nodes.

Gene expression profiles of clonally expanded CD4+ T cells from lymph nodes collected before and after GAD injection, highlighting transcriptional changes associated with antigen-driven activation.

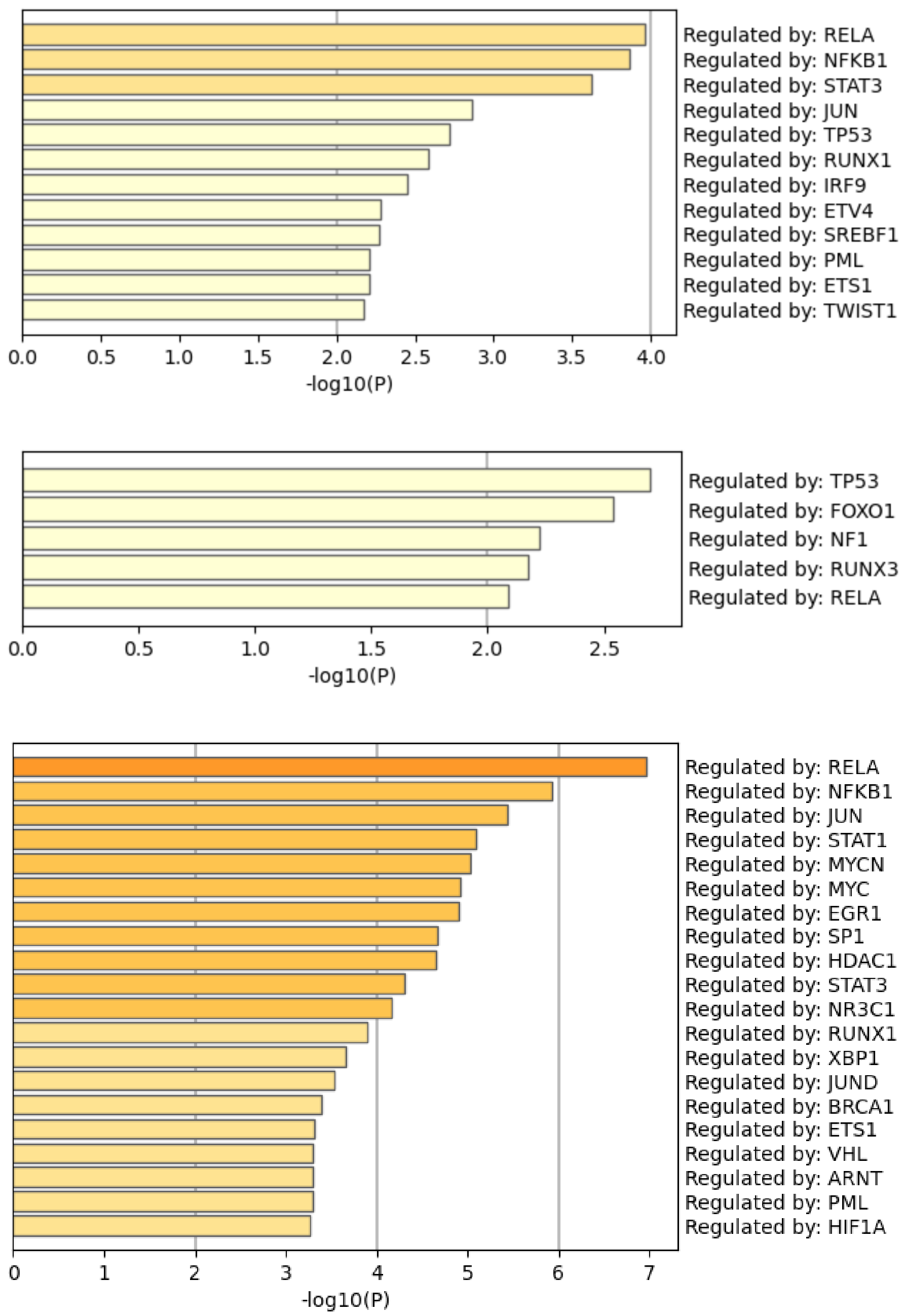

Supplemental Figure 9: TRRUST Analysis of pseudobulk scRNAseq A. Genes upregulated in clonal CD4+ T cells in the post-GAD LN compared to the pre-GAD LN. B Genes upregulated in clonal CD8+ T cells in the post-GAD LN compared to the pre-GAD LN. C. Genes upregulated in CD4+ T cells in the *in vitro* GAD-stimulated PBMC compared to the Ctrl stimulation.

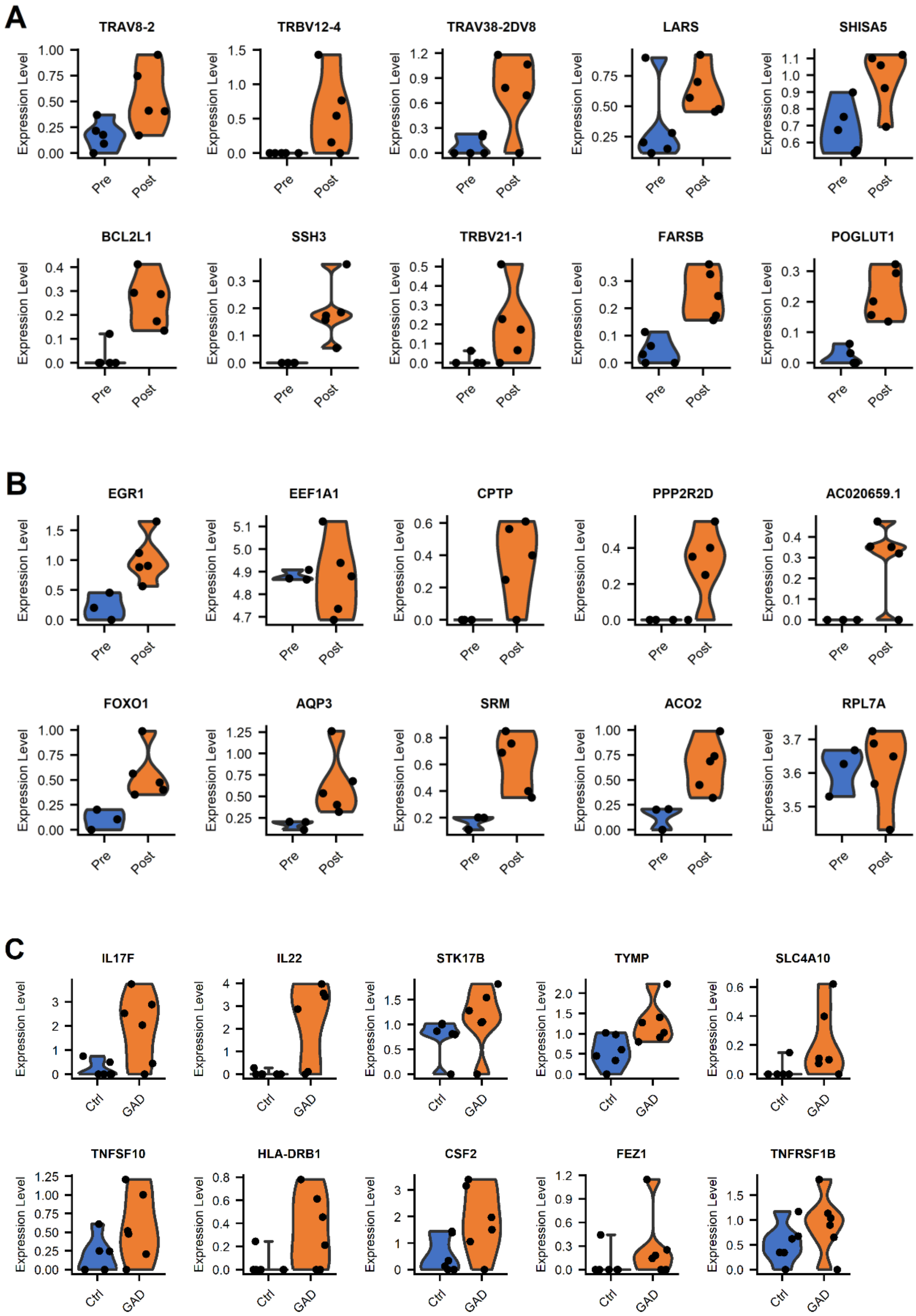

Supplemental Figure 10: Top ten upregulated genes in pseudobulk analysis. A. In clonally expanded CD4+ T cells in the Post_GAD LN compared to the Pre_GAD LN. B. In clonally expanded CD8+ T cells in the Post_GAD LN compared to the Pre_GAD LN. C. In GAD-stimulated PBMC vs Ctrl stimulated PBMC. Each donor is shown as a individual black dot.

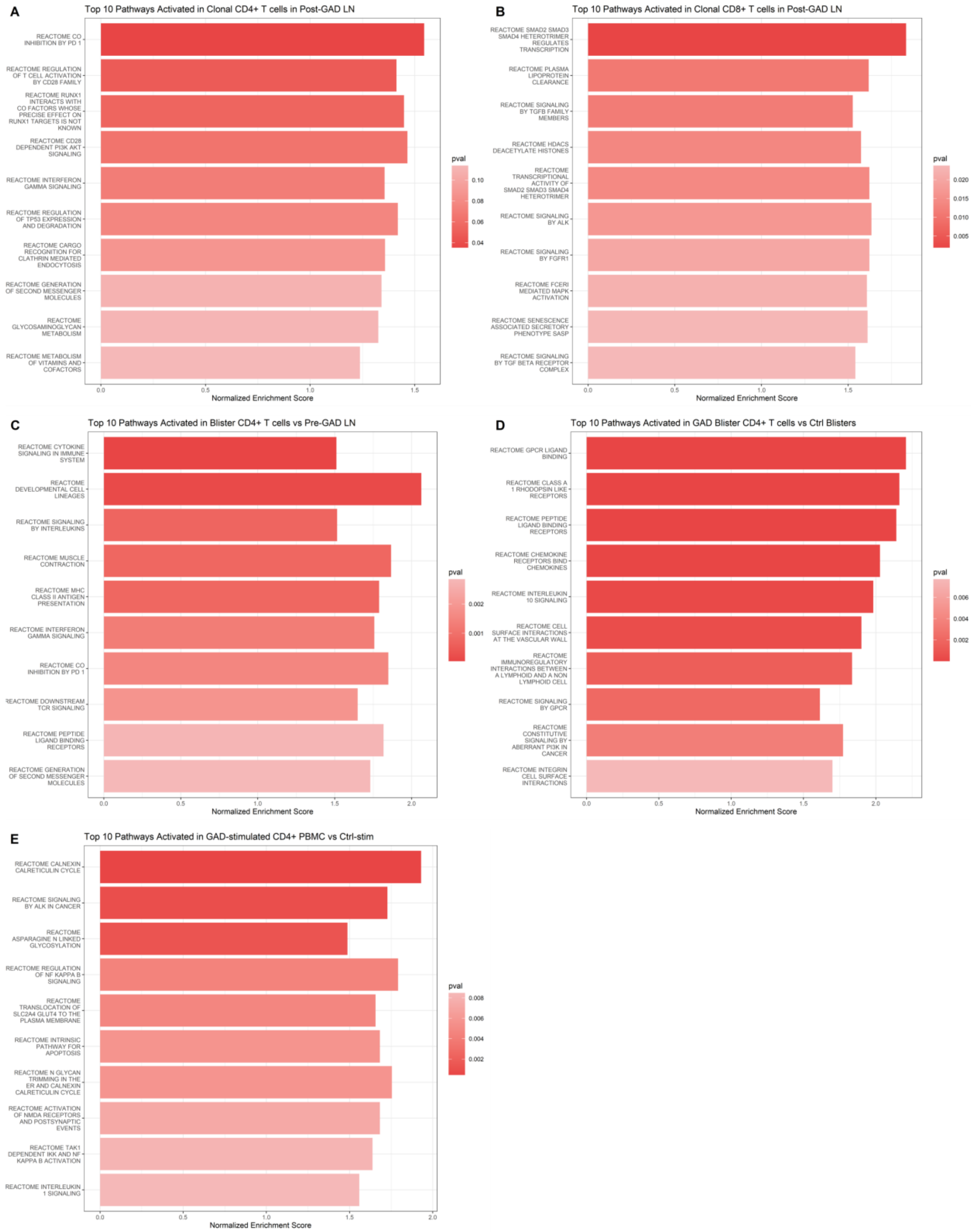

Supplemental Figure 11: GSEA of scRNAseq

Upregulated genes in the indicated samples were clustered into Reactome Pathways and the top ten pathways are shown with a normalized enrichment score and associated p value.

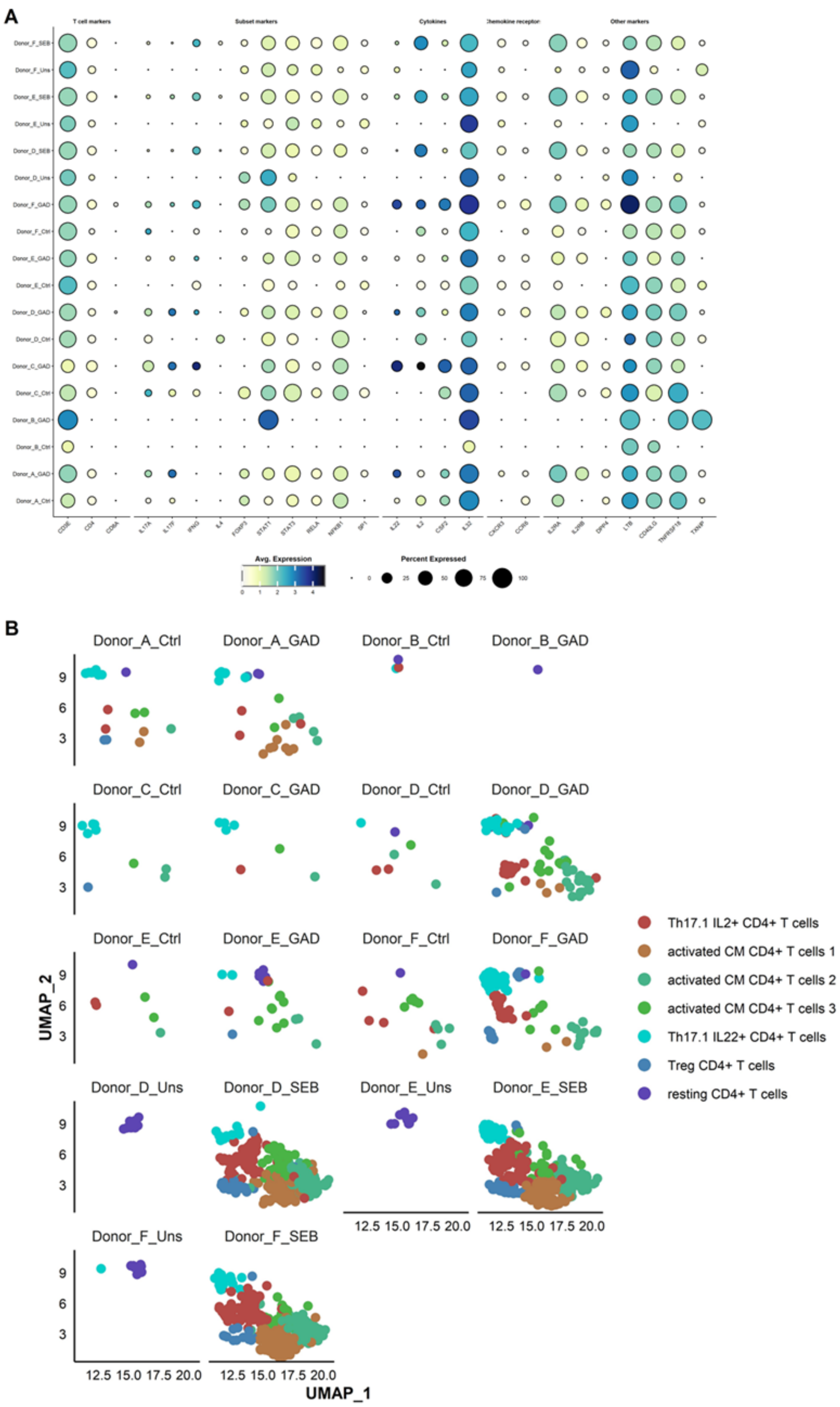

Supplemental Figure 12. Gene expression and clustering of PBMCs following in vitro stimulation.

A. Dot plot showing gene expression profiles of PBMCs, split by donor and stimulation condition. **B**. UMAP plots of all cells, split by donor and in vitro stimulation condition, illustrating clustering patterns across experimental groups.

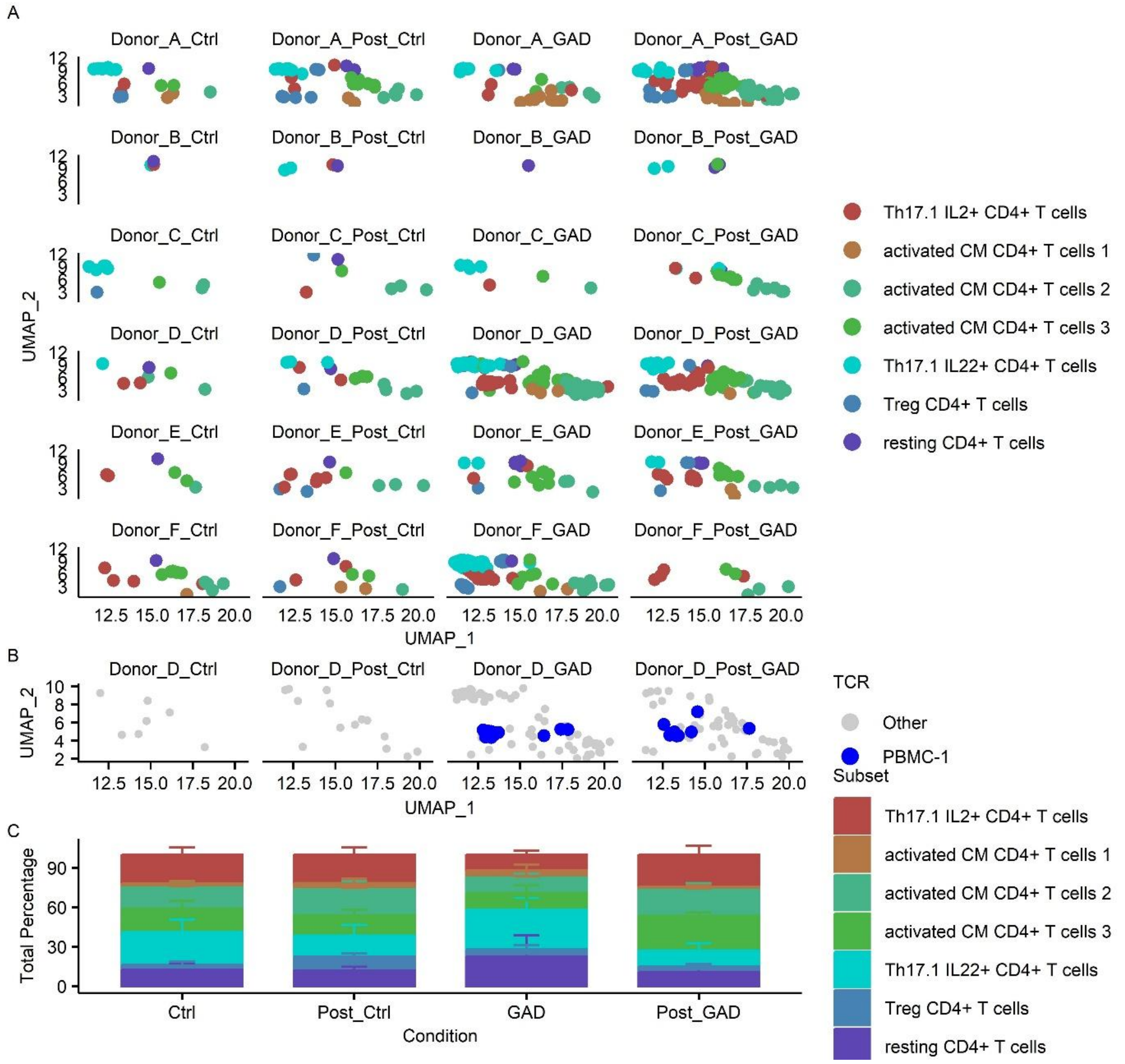

Supplemental Figure 13: Analysis of *in vitro* GAD-stimulated and Ctrl-stimulated CD4+ T cells, Pre and Post *In vivo* GAD-Alum. PBMC were taken from donors Post_GAD and stimulated at the same time as the pre *in vivo* GAD samples. Activated cells were sorted and subjected to scRNAseq. A UMAP plots of all CD4+ T cells after QC, split by donor, *in vitro* stimulation (Ctrl/GAD) and *in vivo* exposure to GAD Alum (NA/Post). B. Distribution of PBMC-1 TCR clones in Donor_D's samples. C. Distribution of CD4+ T cells across subsets, split by *in vitro* stimulation and *in vivo* exposure to GAD Alum

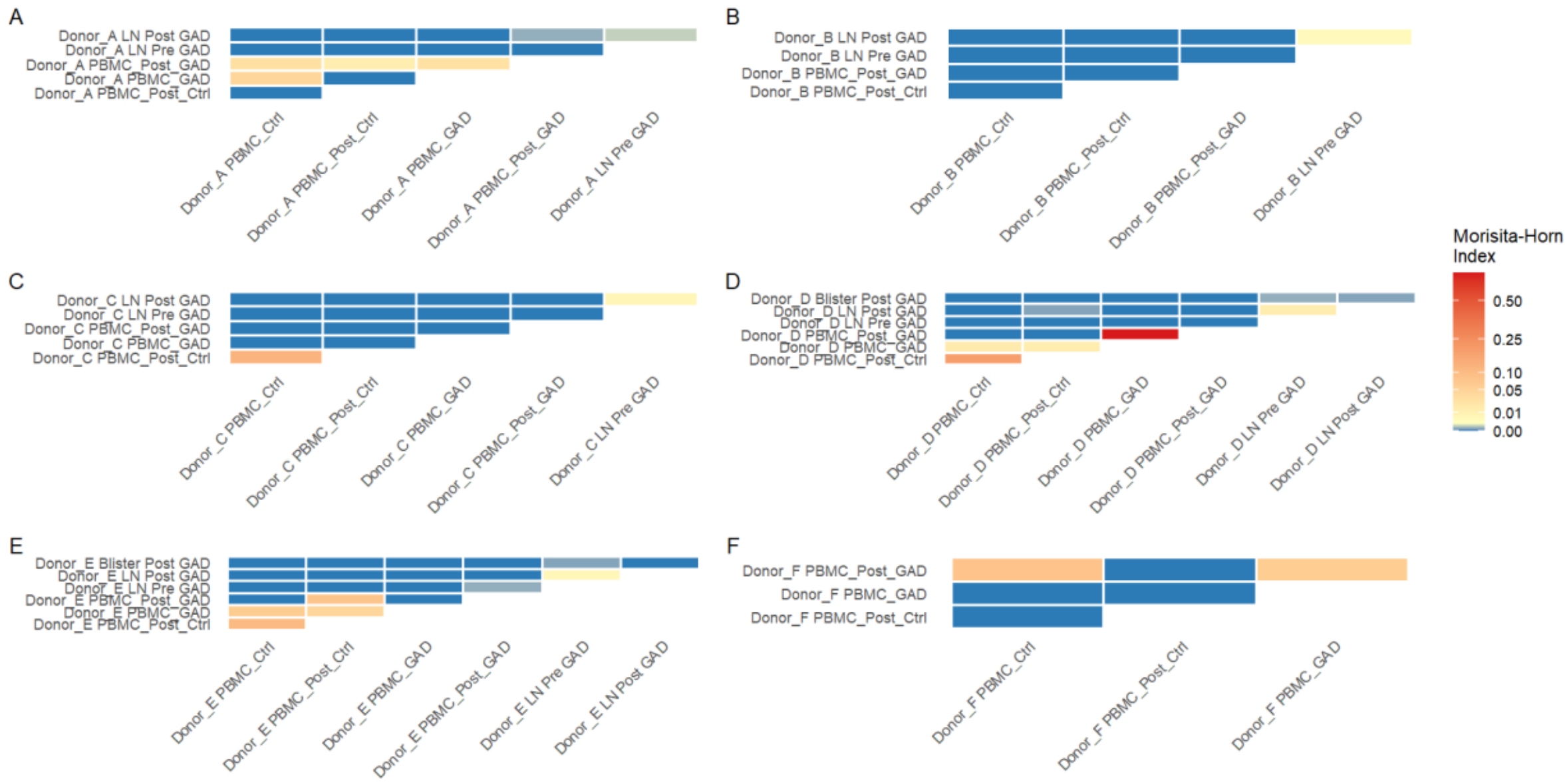

Supplemental Figure 14: Morisita-Horn Similarity Index. The Morisita-Horn similarity index was calculated for paired TCRs for each sample from the Seurat objects after QC. This is a metric of the proportion of shared TCRs between two samples.

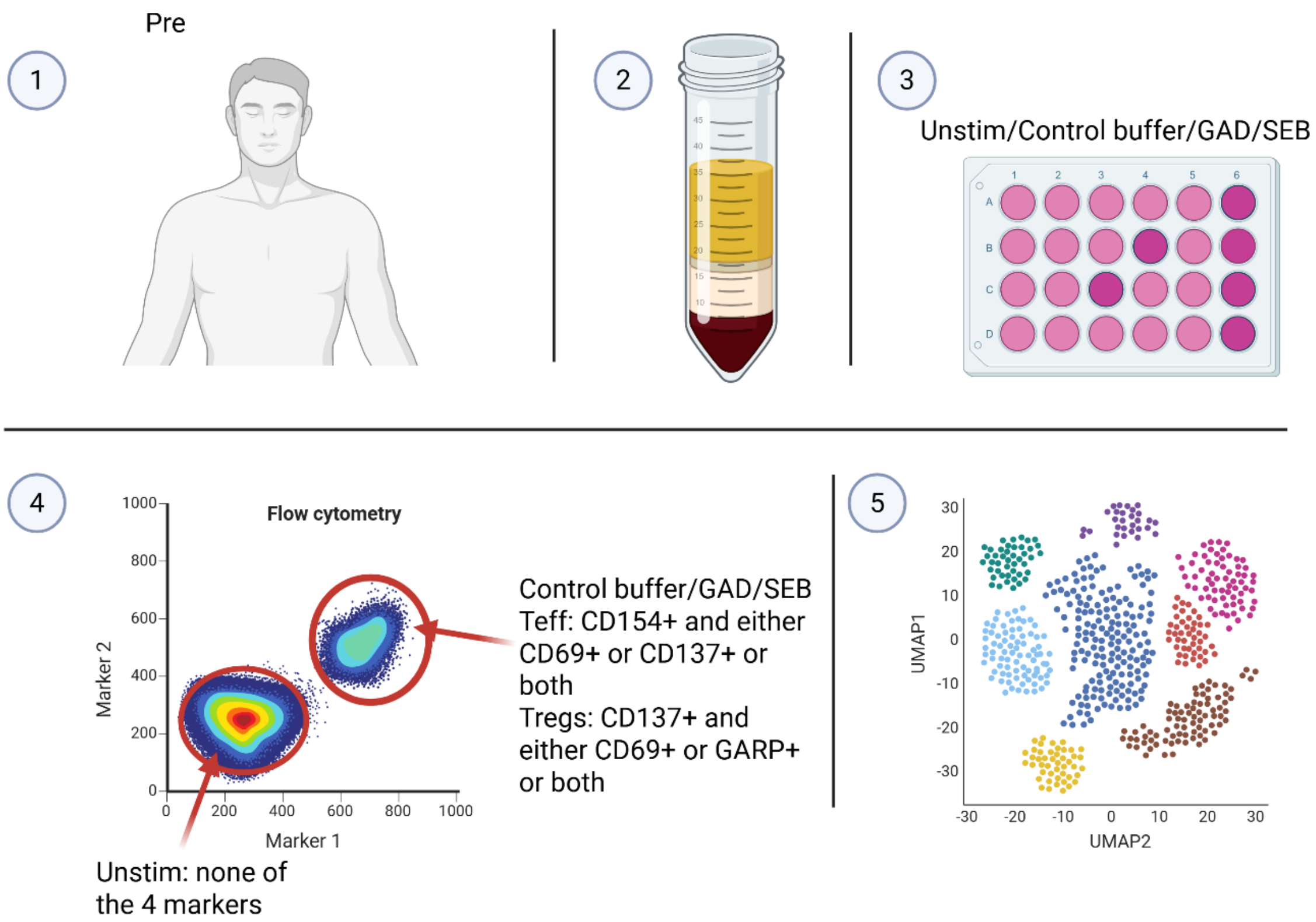

Supplemental Figure 15: Outline of the AIM assay

1. Participants were sampled before *in vivo* injection of GAD-Alum. **2**. Peripheral blood mononuclear cells (PBMCs) were isolated and cryopreserved until required. **3**. PBMCs were thawed and cultured for 18h under one of four conditions. *Unstim*- unstimulated cells cultured in media alone. *Control Buffer* – cells cultured with buffer only. *GAD* – cells cultured with GAD in buffer. *SEB*- cells cultured with the superantigen SEB. **4**. From the Unstim wells, unactivated CD4+ T cells were sorted (negative for all four activation markers). From Control buffer/GAD and SEB wells, activated CD4+ effector T cells (CD154+ and either CD69+ or CD137+ or both) and activated CD4+Tregs (CD137+ and either CD69+ or GARP+ or both) were isolated. The detailed flow cytometry gating strategy is shown in Supplemental Figure 2. **5**. Sorted cells were subjected to scRNAseq. For Supplemental Figure 13 the same procedure was followed but for PBMC sampled post *in vivo* intradermal injection of GAD Alum.

Created in BioRender. H, S. (2026) https://BioRender.com/45z5gll

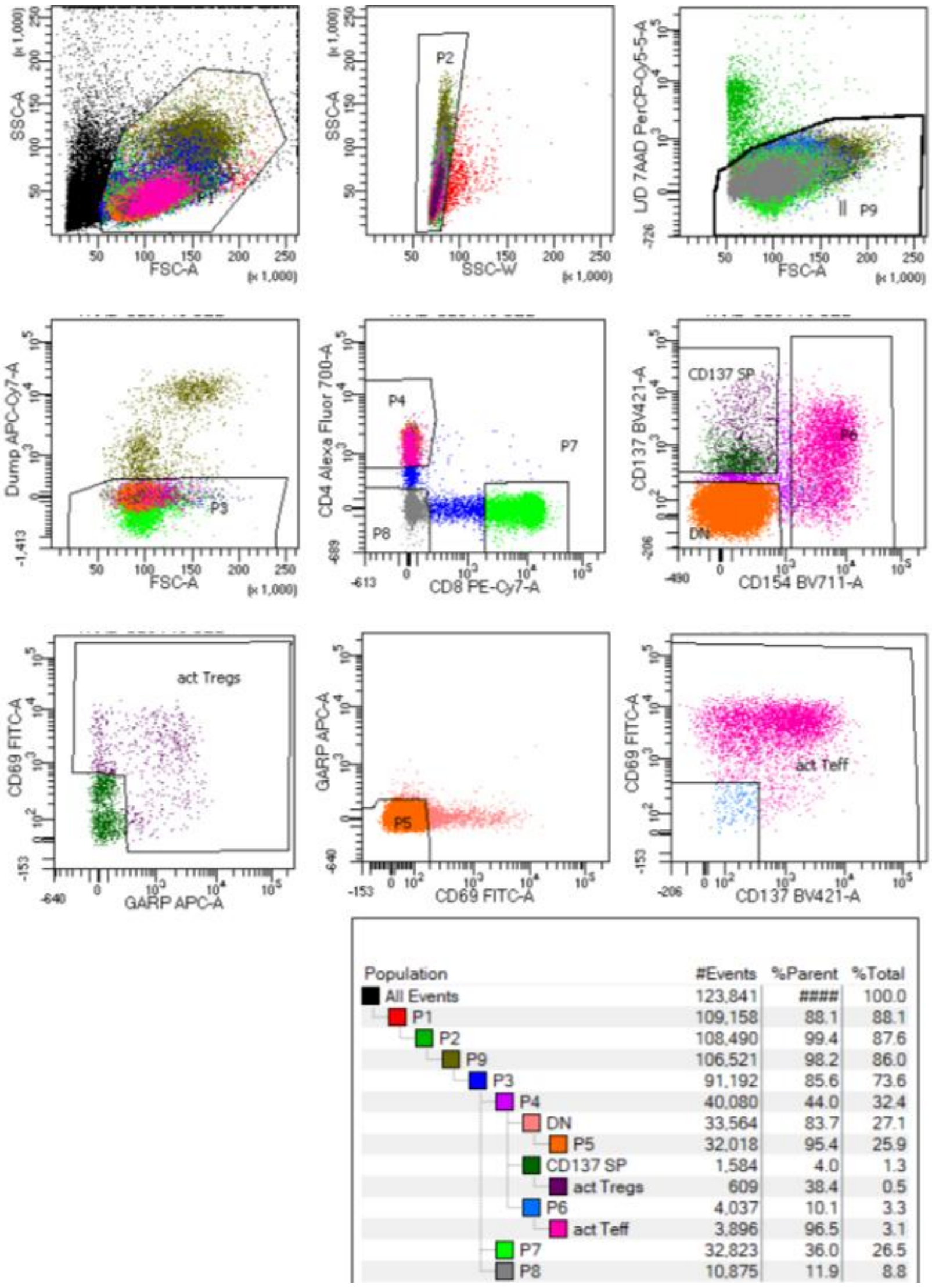

Population	#Events	%Parent	%Total
All Events	123,841	####	100.0
P1	109,158	88.1	88.1
P2	108,490	99.4	87.6
P9	106,521	98.2	86.0
P3	91,192	85.6	73.6
P4	40,080	44.0	32.4
DN	33,564	83.7	27.1
P5	32,018	95.4	25.9
CD137 SP	1,584	4.0	1.3
act Tregs	609	38.4	0.5
P6	4,037	10.1	3.3
act Teff	3,896	96.5	3.1
P7	32,823	36.0	26.5
P8	10,875	11.9	8.8

Supplemental Figure 16. Flow cytometry gating strategy (SEB-stimulated example). Representative gating strategy used to identify activated CD4+ T-cell subsets following SEB stimulation. Shown are sequential gates for live singlet lymphocytes, CD3+CD4+ T cells, and activated effector or regulatory populations based on expression of CD154, CD69, CD137 and GARP. This gating strategy was applied consistently to all stimulation conditions (Unstim, Control buffer, GAD and SEB) to define activated CD4+ Teff and Treg subsets for subsequent single-cell RNA sequencing.
